# Structural and functional interactions between the Ca^2+^-, ATP-, and caffeine-binding sites of skeletal muscle ryanodine receptor (RyR1)

**DOI:** 10.1016/j.jbc.2021.101040

**Published:** 2021-08-02

**Authors:** Venkat R. Chirasani, Daniel A. Pasek, Gerhard Meissner

**Affiliations:** Department of Biochemistry and Biophysics, University of North Carolina, Chapel Hill, North Carolina, USA

**Keywords:** calcium, ATP, AMPPCP, caffeine, allosteric communications, molecular dynamics simulations, ryanodine receptor, sarcoplasmic reticulum, skeletal muscle, AMPPCP, phosphomethylphosphonic acid adenylate ester, ATP/Caf, ATP and caffeine, Caf, caffeine, CSol, core α-solenoid, CTD, C-terminal domain, EC, excitation–contraction, MD, molecular dynamics, MH, malignant hyperthermia, PDB, Protein Data Bank, RyR, ryanodine receptor, RyR1, ryanodine receptor type 1, SASA, solvent-accessible surface area, SR, sarcoplasmic reticulum, TaF, thumb and forefingers

## Abstract

Ryanodine receptor type 1 (RyR1) releases Ca^2+^ ions from the sarcoplasmic reticulum of skeletal muscle cells to initiate muscle contraction. Multiple endogenous and exogenous effectors regulate RyR1, such as ATP, Ca^2+^, caffeine (Caf), and ryanodine. Cryo-EM identified binding sites for the three coactivators Ca^2+^, ATP, and Caf. However, the mechanism of coregulation and synergy between these activators remains to be determined. Here, we used [^3^H]ryanodine ligand-binding assays and molecular dynamics simulations to test the hypothesis that both the ATP- and Caf-binding sites communicate with the Ca^2+^-binding site to sensitize RyR1 to Ca^2+^. We report that either phosphomethylphosphonic acid adenylate ester (AMPPCP), a nonhydrolyzable ATP analog, or Caf can activate RyR1 in the absence or the presence of Ca^2+^. However, enhanced RyR1 activation occurred in the presence of Ca^2+^, AMPPCP, and Caf. In the absence of Ca^2+^, Na^+^ inhibited [^3^H]ryanodine binding without impairing RyR1 activation by AMPPCP and Caf. Computational analysis suggested that Ca^2+^-, ATP-, and Caf-binding sites modulate RyR1 protein stability through interactions with the carboxyterminal domain and other domains in the activation core. In the presence of ATP and Caf but the absence of Ca^2+^, Na^+^ is predicted to inhibit RyR1 by interacting with the Ca^2+^-binding site. Our data suggested that ATP and Caf binding affected the conformation of the Ca^2+^-binding site, and conversely, Ca^2+^ binding affected the conformation of the ATP- and Caf-binding sites. We conclude that Ca^2+^, ATP, and Caf regulate RyR1 through a network of allosteric interactions involving the Ca^2+^-, ATP-, and Caf-binding sites.

Ryanodine receptors (RyRs) release Ca^2+^ ions from the sarcoplasmic reticulum (SR), an intracellular Ca^2+^-storing compartment, to initiate muscle contraction (1, 2, 3). RyRs are comprised of four 560 kDa subunits with a total molecular weight of 2250 kDa. There are three RyR isoforms in mammalian cells: ryanodine receptor type 1 (RyR1) predominantly expressed in skeletal muscle, RyR2 majorly expressed in cardiac muscle, and RyR3 expressed in multiple tissues but at lower concentrations. Multiple endogenous and exogenous effectors regulate the RyRs. RyR1 is controlled by direct interaction with Ca_v_1.1 voltage-gated Ca^2+^ channels and by Ca^2+^ through a mechanism not well understood (2, 3). In contrast, influx of extracellular Ca^2+^ through voltage-gated Ca^2+^ channels activate RyR2 and RyR3, which triggers the release of Ca^2+^ through a Ca^2+^-induced Ca^2+^ release mechanism. Micromolar Ca^2+^ and millimolar ATP activate the RyRs and millimolar Ca^2+^ and millimolar Mg^2+^ inhibit the RyRs (2). Among the large number of exogenous ligands, caffeine (Caf) has been extensively used to assess the role of RyRs in controlling cellular Ca^2+^ concentrations in normal muscle and human muscle pathologies (4). Studies with skinned muscle fibers, isolated membrane preparations, and purified RyRs indicate that Caf causes the release of Ca^2+^ by activating RyRs. Exposure of skeletal muscle RyR1 missense mutations associated with malignant hyperthermia (MH) to Caf results in the release of large quantities of Ca^2+^ from the SR, which causes a rapid rise in body temperature and severe muscle contractions (5, 6).

Structural analysis by crystallography and cryo-EM has resolved the structure of the homotetrameric RyR1 and RyR2 protein complexes at near-atomic resolution (7, 8, 9, 10, 11, 12, 13, 14, 15). As predicted by earlier studies, approximately 500 amino acids of the RyR carboxyl terminal domain constitute the transmembrane domain and cation-conducting pore. The remaining ∼90% of RyRs form the cytoplasmic domain comprised of the activation core harboring Ca^2+^-, ATP-, and Caf-binding sites, and a large cytoplasmic shell serving as a target for calmodulin, protein kinases, and redox active species regulating RyR1 *via* long-range allosteric mechanisms (Fig. 1).

**Figure 1:**
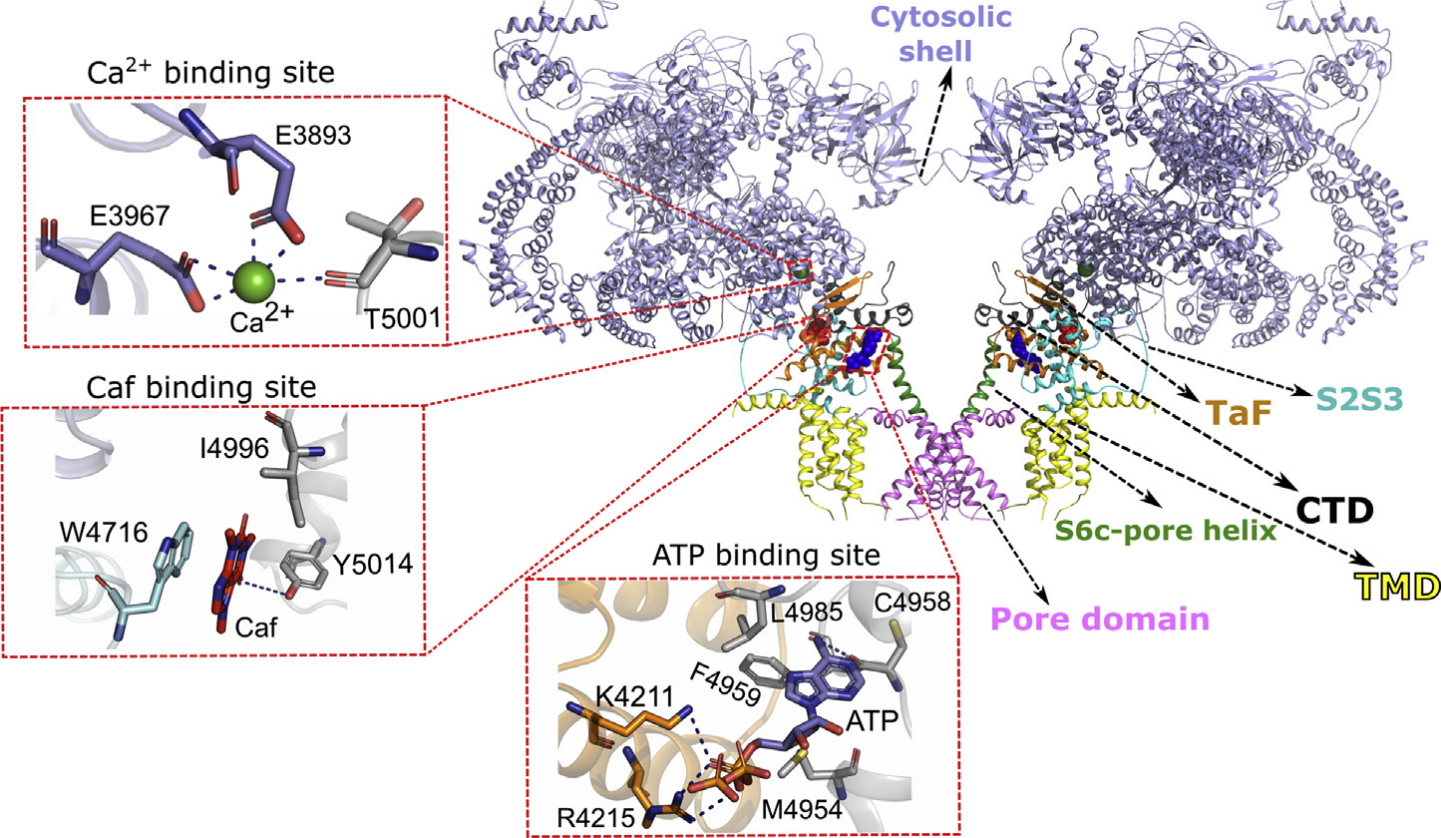
**RyR1 architecture and domain organization.** 3D conformation of RyR1 + ATP/Caf/Ca^2+^ (*open*) (Protein Data Bank: 5TAL) with Ca^2+^-, ATP-, and Caf-binding sites. Two of four chains shown depict the activation core and ligand sites. The color code of individual domains is as follows: cytosolic shell (*light blue*), S2S3 domain (*cyan*), TaF domain (*orange*), S6c-pore helix (*green*), pore domain (*magenta*), TMD (*yellow*), and CTD (*black*). Binding sites for Ca^2+^ (*green*), ATP (*blue*), and Caf (*red*) are enlarged to show specific interactions for each ligand. The activation ligands are in the activation core and at the interfaces of CTD, CSol, S2S3, TaF, and S6c domains. Caf, caffeine; CMD, COOH-terminal membrane-anchoring domain; CSol, core α-solenoid domain; CTD, C-terminal domain; RyR1, ryanodine receptor type 1; TMD, transmembrane domain.

RyR1 exhibits open and closed states in the presence of three activators—Ca^2+^, ATP, and Caf. Cryo-EM data suggested that Ca^2+^ binds between core α-solenoid (CSol) domain and the C-terminal domain (CTD), Caf binds at the interface of S2S3 and the CTD, and ATP binds at the junction of thumb and forefingers (TaF), S6c, and the CTD domains (Fig. 1). Although Caf is a known modulator of RyR1 and binds at the interface of the CTD, S2S3, and TaF domains, it is still unknown what occupies this site under normal physiological conditions. The addition of either Ca^2+^ alone or ATP and Caf (ATP/Caf) together reduced channel open probabilities and restrained open channel states in cryo-EM (11). Single-channel experiments revealed that a combination of micromolar Ca^2+^ and millimolar ATP would be sufficient to open the RyR1 channel. Furthermore, the addition of RyR1 channel activator—Caf—has maximized the channel open probability. Murayama *et al.* (16) reported that the RyR2 Caf-binding site regulates Ca^2+^ sensitivity through an interaction between two hydrophobic residues, RyR2-Try-4644 and RyR2-Ile-4925 (corresponding to conserved RyR1-Trp-4716 and RyR1-Ile-4996). Furthermore, in the presence of ATP, the binding of Caf breaks interactions between CTD and S2S3 domains and rearranges Ca^2+^-binding pocket for favorable Ca^2+^ binding. This suggests that the binding of ATP and Caf modulates the Ca^2+^-binding site and affect Ca^2+^ sensitivity of RyR1 (2). Furthermore, the proximal location of Ca^2+^-, ATP-, and Caf-binding sites within the activation domain suggests coordinated communication between the ligand-binding sites. However, it remains unclear how ATP- and Caf-binding sites communicate with the Ca^2+^-binding site to sensitize RyR1 to Ca^2+^.

In the present study, we employed biochemical and molecular dynamics (MD) simulations to test the hypothesis that both the ATP- and Caf-binding sites communicate with the Ca^2+^-binding site to sensitize RyR1 to Ca^2+^. A [^3^H]ryanodine ligand-binding method was used to determine the regulation of RyR1 by Ca^2+^, phosphomethylphosphonic acid adenylate ester (AMPPCP), a nonhydrolyzable ATP analog, and Caf. The structures of Ca^2+^-, ATP-, and Caf-binding sites were resolved using cryo-EM micrograph densities of purified RyR1 in the presence of 0.5 M NaCl (11). Our studies were performed in 0.5 M choline chloride (Cl) media to eliminate potential interactions of Na^+^ with the Ca^2+^-binding site (17). We report that Caf or AMPPCP activated RyR1 in the absence and presence of Ca^2+^, and an amplified RyR1 activation is evident in the presence of AMPPCP and Caf (AMPPCP/Caf). To determine the effects of Na^+^ on RyR1 activity, [^3^H]ryanodine binding to RyR1 was compared in 0.5 M choline Cl or 0.5 M NaCl media. Computational methods using cryo-EM densities suggest that RyR1 is regulated through a network of allosteric interactions involving the Ca^2+^-, ATP-, and Caf-binding sites.

## Results

### Regulation of RyR1 by Ca^2+^, ATP, and Caf in choline Cl medium

Cryo-EM provides insights in the structural modulation of the Ca^2+^activation site of RyR1 by Ca^2+^ and the combined action of ATP and Caf. However, it is not known how ATP or Caf in the absence of the other coactivator modulates the structure of the Ca^2+^-binding site. In the present study, the Ca^2+^-dependent regulation of RyR1 was determined in the absence and presence of Caf, AMPPCP, and the presence of both ligands, where enhanced binding correlates with an increased open channel probability (*P*_o_) in lipid bilayers (2). [^3^H]Ryanodine binding levels were determined in 0.5 M choline Cl media, taking advantage of the observation that elevated concentrations of Cl^−^ activate RyR1, and the use of choline^+^ eliminates the inhibitory action of Na^+^ on the Ca^2+^ activation site (17). To assess the effects of Na^+^, we determined the [^3^H]ryanodine binding levels of RyR1 in 0.5 M NaCl media.

**Figure 2:**
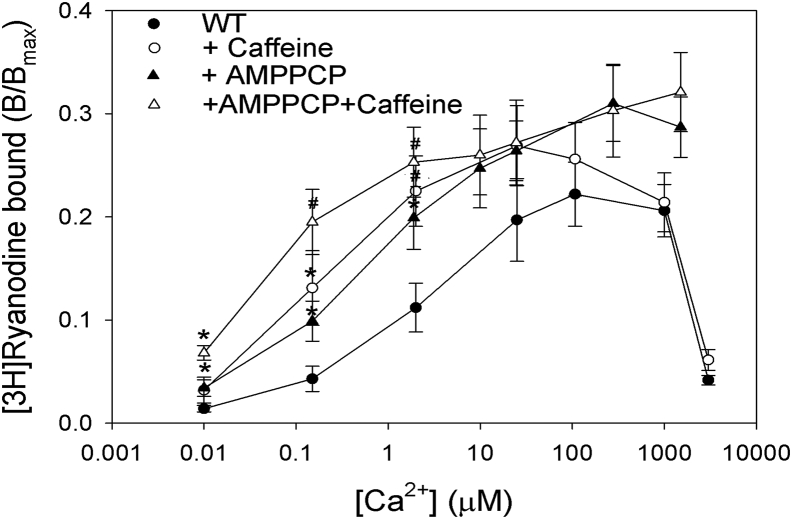
**[**^**3**^**H]Ryanodine binding of RyR1 in the presence of increasing Ca**^**2+**^**concentrations with and without AMPPCP and caffeine.** Specific [^3^H]ryanodine binding to RyR1 was determined in 0.5 M choline Cl, 20 mM imidazole, pH 7 with 2 nM [^3^H]ryanodine and the indicated Ca^2+^ in the absence or the presence of 2 mM AMPPCP and/or 5 mM caffeine. Data are the mean ± SE of four to seven experiments. ^#^*p* < 0.05 compared with Ca^2+^ only by two-way ANOVA followed by Holm–Sidak method and ∗*p* < 0.05 by Student's *t* test. RyR1, ryanodine receptor type 1.

Figure 2 shows that addition of 2 mM AMPPCP or 5 mM Caf increased [^3^H]ryanodine binding at free Ca^2+^ concentrations from nominally 0.01 to 100 μM Ca^2+^ in 0.5 M choline Cl. An additional increase in [^3^H]ryanodine binding was observed in the combined presence of AMPPCP/Caf. At Ca^2+^ concentrations exceeding 100 μM, AMPPCP further increased [^3^H]ryanodine binding in the presence or absence of Caf, whereas Caf alone did not show any effect. At 0.01 μM Ca^2+^, AMPPCP and the combined presence of AMPPCP/Caf significantly increased [^3^H]ryanodine binding. At 0.15 μM Ca^2+^, [^3^H]ryanodine binding was significantly increased by AMPPCP/Caf and at 2 μM Ca^2+^ by Caf and AMPPCP/Caf as determined by two-way ANOVA. The results suggest that AMPPCP/Caf independently regulated the activity of RyR1 in the absence or presence of Ca^2+^.

### Regulation of RyR1 in choline Cl and NaCl media

**Figure 3:**
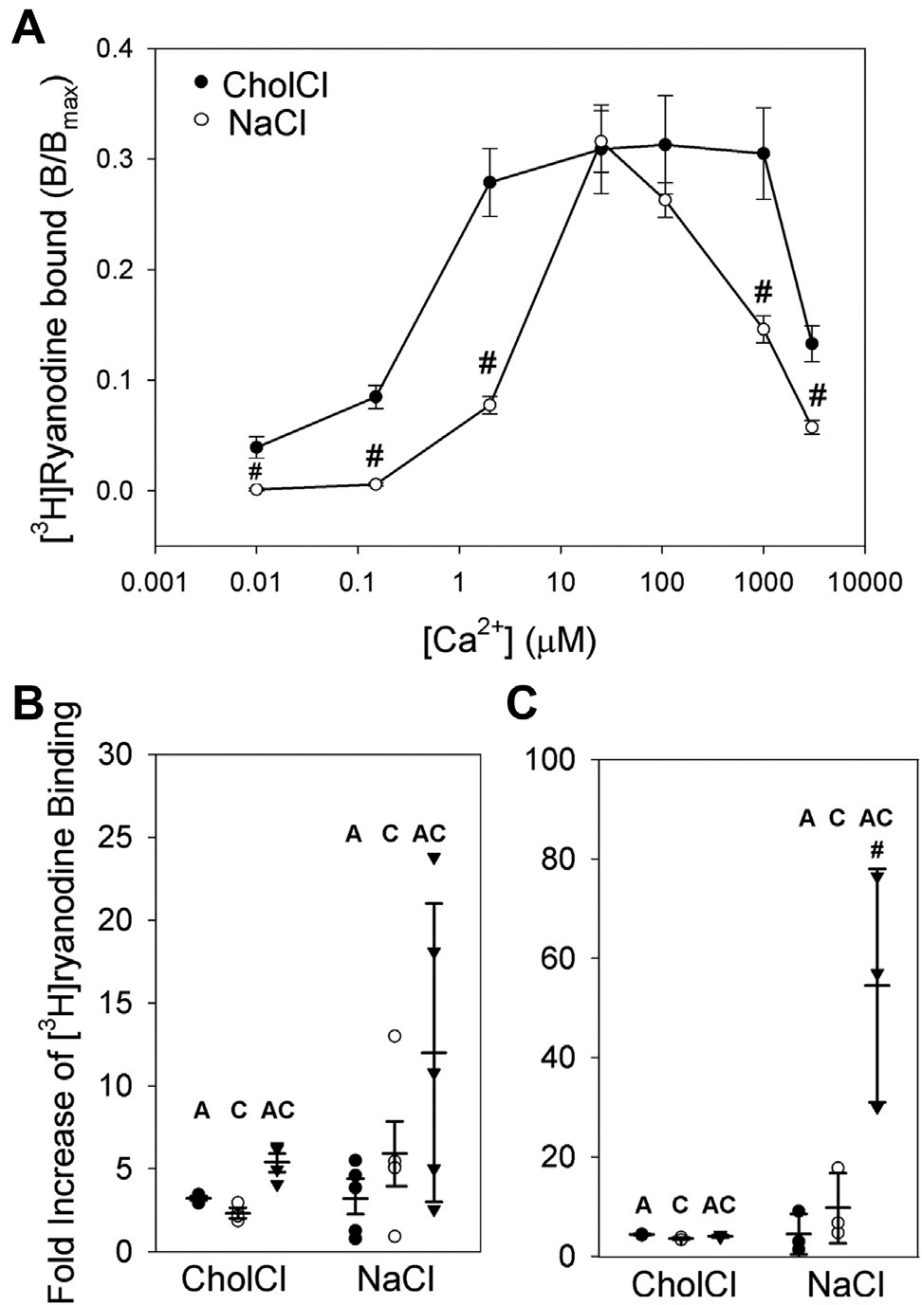
**[**^**3**^**H]Ryanodine binding of RyR1 in choline chloride (choline Cl) and NaCl media.***A*, specific [^3^H]ryanodine binding to RyR1 was determined in 0.5 M choline Cl or 0.5 M NaCl, 20 mM imidazole, pH 7 with 2 nM [^3^H]ryanodine and the indicated Ca^2+^ in the absence and presence of 2 mM AMPPCP and/or 5 mM caffeine (Caf). *B* and *C*, specific [^3^H]ryanodine binding to RyR1 was determined in 0.5 M choline Cl or 0.5 M NaCl, 20 mM imidazole, pH 7 with 5 nM [^3^H]ryanodine and nominally 0.01 μM Ca^2+^ (*B*) and 25 μM Ca^2+^ (*C*) before and after the addition of 2 mM AMPPCP and/or 5 mM Caf. Data are the mean ± SE of four to five experiments (*B*) and mean ± SD of three experiments (*C*). #*p* < 0.05 compared with Ca^2+^ only by two-way ANOVA followed by Holm–Sidak method. Labels used in the figure: A refers to ATP, C refers to Caf, and AC refers to ATP + Caf. RyR1, ryanodine receptor type 1.

des Georges *et al.* (11) determined the structures of the Ca^2+^-, ATP-, and Caf-binding sites of purified RyR1s in 0.5 M NaCl. To assess the effects of Na^+^ on RyR1 activity, we compared [^3^H]ryanodine binding to RyR1 in 0.5 M choline Cl or 0.5 M NaCl media at free Ca^2+^ concentrations ranging from 10 nM to 3 mM. Figure 3*A* shows that in choline Cl medium, RyR1 displayed a biphasic Ca^2+^ activation/inhibition profile with maximal activity ranging from ∼2 to ∼100 μM and in NaCl medium with maximal activity at ∼20 μM Ca^2+^. At 0.01 μM Ca^2+^, significantly lower [^3^H]ryanodine binding was observed in 0.5 M NaCl at 0.01 μM, 0.15 μM, 2 μM, 1 mM, and 3 mM Ca^2+^ by two-way ANOVA compared with 0.5 M choline Cl.

The addition of AMPPCP/Caf to 0.5 M NaCl media increased [^3^H]ryanodine binding. At 0.01 μM Ca^2+^, no significant differences were observed between fold increased [^3^H]ryanodine-binding levels in 0.5 M NaCl compared with 0.5 M choline Cl with the addition of AMPPCP, Caf, or AMPPCP/Caf (Fig. 3*B*). At 25 μM Ca^2+^, the combined addition of AMPPCP/Caf significantly increased [^3^H]ryanodine binding, whereas RyR1 activity did not increase in the presence of AMPPCP or Caf in 0.5 M NaCl compared with 0.5 M choline (Fig. 3*C*).

### RyR1 pore structure and stability

The effects of Ca^2+^, ATP, and Caf on RyR1 pore structure and stability were modeled. Computational analysis of cryo-EM densities of purified RyR1 in 0.5 M NaCl suggested a closed pore structure for the apo-RyR1, RyR1 + Ca^2+^, and RyR1 + ATP/Caf states (11). About equal portions of the RyR1 + ATP/Caf/Ca^2+^ exhibited a closed and open pore structure. We confirmed that in our MD simulations with choline Cl media, RyR1 maintained a closed pore structure for the apo-RyR1 (Protein Data Bank [PDB]: 5TB0), RyR1 + Ca^2+^ (PDB: 5T15), and RyR1 + ATP/Caf (PDB: 5TAP) states (Table S1 and Fig. S1). We have also observed closed and open pore structures for RyR1 + ATP/Caf/Ca^2+^ state (Table S1 and Fig. S1) consistent with the cryo-EM data (11). To understand the role of ATP and Caf on RyR1 pore conformation and Ca^2+^-binding site, we modeled and simulated RyR1 + ATP/Ca^2+^ and RyR1 + Caf/Ca^2+^ by considering open RyR1 + ATP/Caf/Ca^2+^ (PDB: 5TAL) as starting structure. The subsequent trajectory analyses depicted an open pore conformation for RyR1 + ATP/Ca^2+^ and closed pore conformation for RyR1 + Caf/Ca^2+^. The comparison of average radius profiles of pore region from MD trajectories with respective starting cryo-EM structures demonstrated significant perturbations in the pore structure (Fig. S2). These alterations in pore region denote domain rearrangements in RyR1 essential for its functionality.

**Figure 6:**
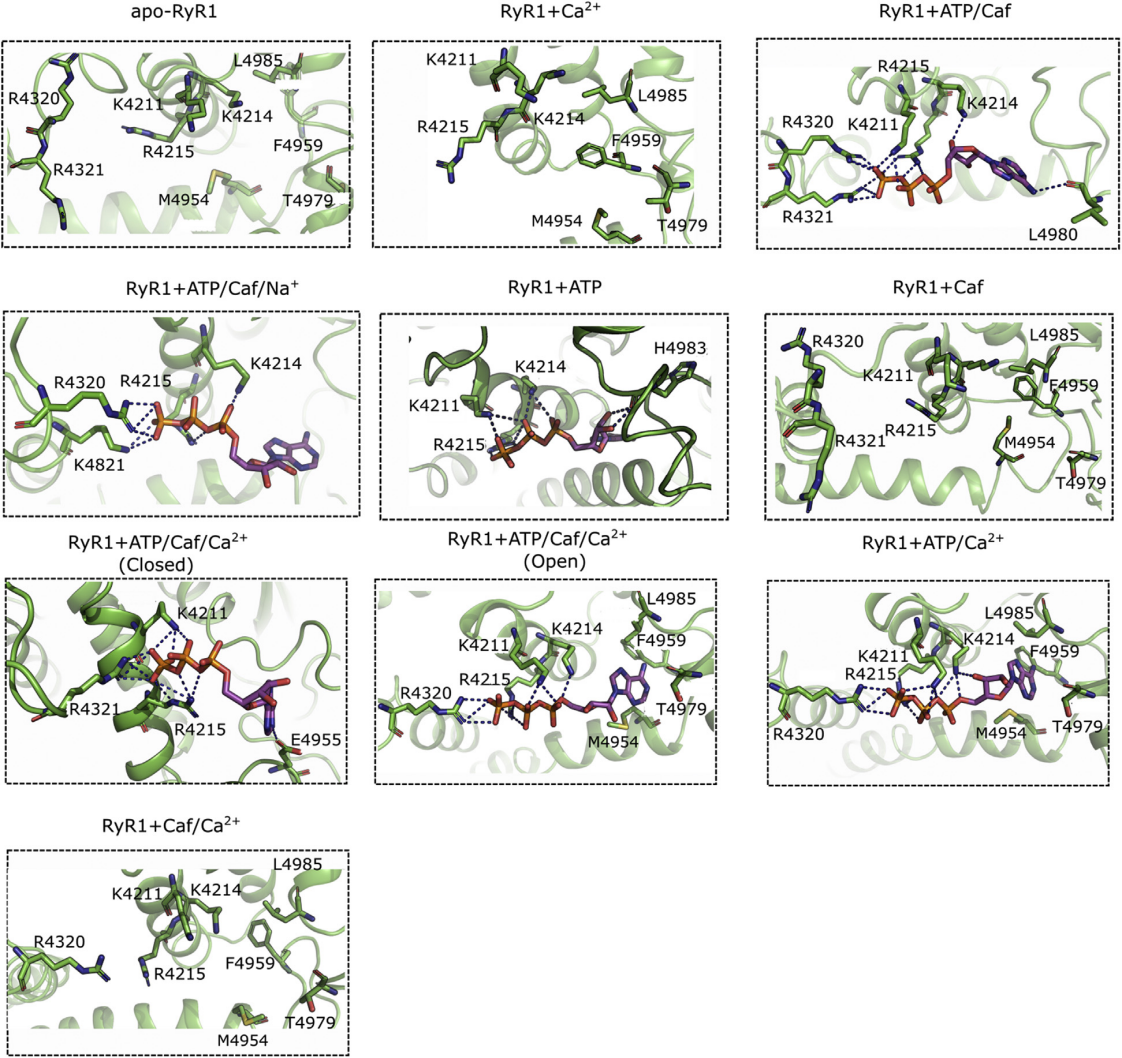
**Evaluation of ATP-binding site interactions in different RyR1 functional states.** Interactions of ATP molecule residues with pocket lining amino acid in different RyR1 functional states were evaluated by extracting average structures from last 20 ns of respective trajectories. Our comparative analysis suggests that ATP has differential interactions in open and closed RyR1 states. Furthermore, the ATP-site interactions were modulated by the presence or absence of Ca^2+^ and/or Caf. The protein is shown as cartoon in *green* with ATP-binding site residues in *stick representation*. ATP molecule is rendered in *magenta* stick representation, and H-bond interactions are shown as *blue dotted lines*. RyR1, ryanodine receptor type 1.

The RMSD of protein residues estimates the stability of RyR1 protein structure (Fig. S3 and Table 1). Comparison of RMSD profiles among the MD systems with RyR1 in different functional states highlights the significance of ligand binding on RyR1 stability. Specifically, the binding of ATP/Caf/Ca^2+^ in open RyR1 conformation induced little or no fluctuations in RyR1 structure (RMSD = 2.5 ± 0.1 Å). On the contrary, RyR1 with closed pore conformation, irrespective of the presence or absence of the ligands, depict large fluctuations ranging between 9.2 ± 1.5 and 10.5 ± 1.1 Å (Fig. S3 and Table 1). Interestingly, the absence of ATP destabilized RyR1 + Caf/Ca^2+^ structure, whereas the absence of Caf stabilized RyR1 + ATP/Ca^2+^ structure in open conformation (RMSD =1.4 ± 0.2). The stable and open RyR1 conformation exhibited by RyR1 + ATP/Ca^2+^ and RyR1 + ATP/Caf/Ca^2+^ systems can be attributed to the polar interactions induced by triphosphate group of ATP (Fig. 6), as previous studies demonstrate that the triphosphate moiety alone can activate RyR2 (18). In addition, the presence of Na^+^ ion in the Ca^2+^-binding site of RyR1 + ATP/Caf reduced RMSD from 10.4 ± 0.7 to 2.4 ± 0.3 and stabilized the RyR1 conformation in a closed state, which further emphasizes the relation between effector binding and RyR1 stability.

**Table 1:** Stability (RMSD) of RyR1 and SASA of Ca^2+^-binding site

MD system name	Ligands	Pore status	RMSD (Å)		Choline Cl	SASA (Å^2^)	
			Choline Cl	NaCl		NaCl	Cryo-EM (+ NaCl)
apo-RyR1	None	Closed	9.2 ± 1.5	9.0 ± 1.0	244.7 ± 21.1	246.7 ± 12.7	163.0
RyR1 + Ca^2+^	Ca^2+^	Closed	10.5 ± 0.6	8.56 ± 0.6	121.0 ± 8.7	122.03 ± 11.1	112.0
RyR1 + ATP/Caf	ATP, Caf	Closed	10.4 ± 0.7	2.4 ± 0.3	200.4 ± 24.3	146.7 ± 10.9	133.0
RyR1 + ATP	ATP	Closed	10.1 ± 1.1	ND	197.5 ± 22.1	ND	ND
RyR1 + Caf	Caf	Closed	9.3 ± 1.4	ND	239.1 ± 20.6	ND	ND
RyR1 + ATP/Caf/Ca^2+^	Ca^2+^, ATP, Caf	Closed	10.5 ± 1.1	ND	147.4 ± 14.1	ND	94.6
RyR1 + ATP/Caf/Ca^2+^	Ca^2+^, ATP, Caf	Open	2.5 ± 0.1	ND	110.0 ± 10.9	ND	82.3
RyR1 + ATP/Ca^2+^	Ca^2+^, ATP	Open	1.4 ± 0.2	ND	70.7 ± 4.9	ND	ND
RyR1 + Caf/Ca^2+^	Ca^2+^, Caf	Closed	10.0 ± 1.4	ND	86.9 ± 9.4	ND	ND

Abbreviation: ND, not determined.

To further probe structural differences among RyR1 in different functional states, we performed domain-wise RMSD analysis (Fig. S4). We evaluated RMSDs of cytosolic shell region and other individual domains of the activation core from each monomer of RyR1. We compared the average RMSD of individual domains among different RyR1 states, in the presence and absence of channel modulators, with respect to simulation time (Fig. S4). The data indicated that minor disturbances in activator binding sites, in the absence of specific activators, induce large fluctuations in cytosolic shell region. The absence of either ATP, Caf, or Ca^2+^ in respective binding sites cause large electrostatic perturbations, which instigate structural changes or disturbances in entire protein. Interestingly, des Georges *et al.* (11) demonstrated that RyR1 exhibits distinct conformations in the absence of activating ligands because of confined motions in the cytosolic region. So, our MD data corroborate well with cryo-EM and experimental findings. However, despite the presence of ATP, Caf, and Ca^2+^ in RyR1 + ATP/Caf/Ca^2+^ (closed; PDB: 5TAQ), the cytosolic shell domain and activation core domains (S2S3, TaF, CTD, CSol, and pore) exhibit larger fluctuations. One possible explanation is the initiation of characteristic changes or movements in the activation module that stimulate the opening of RyR1 channel.

### Ca^2+^-binding site

**Figure 4:**
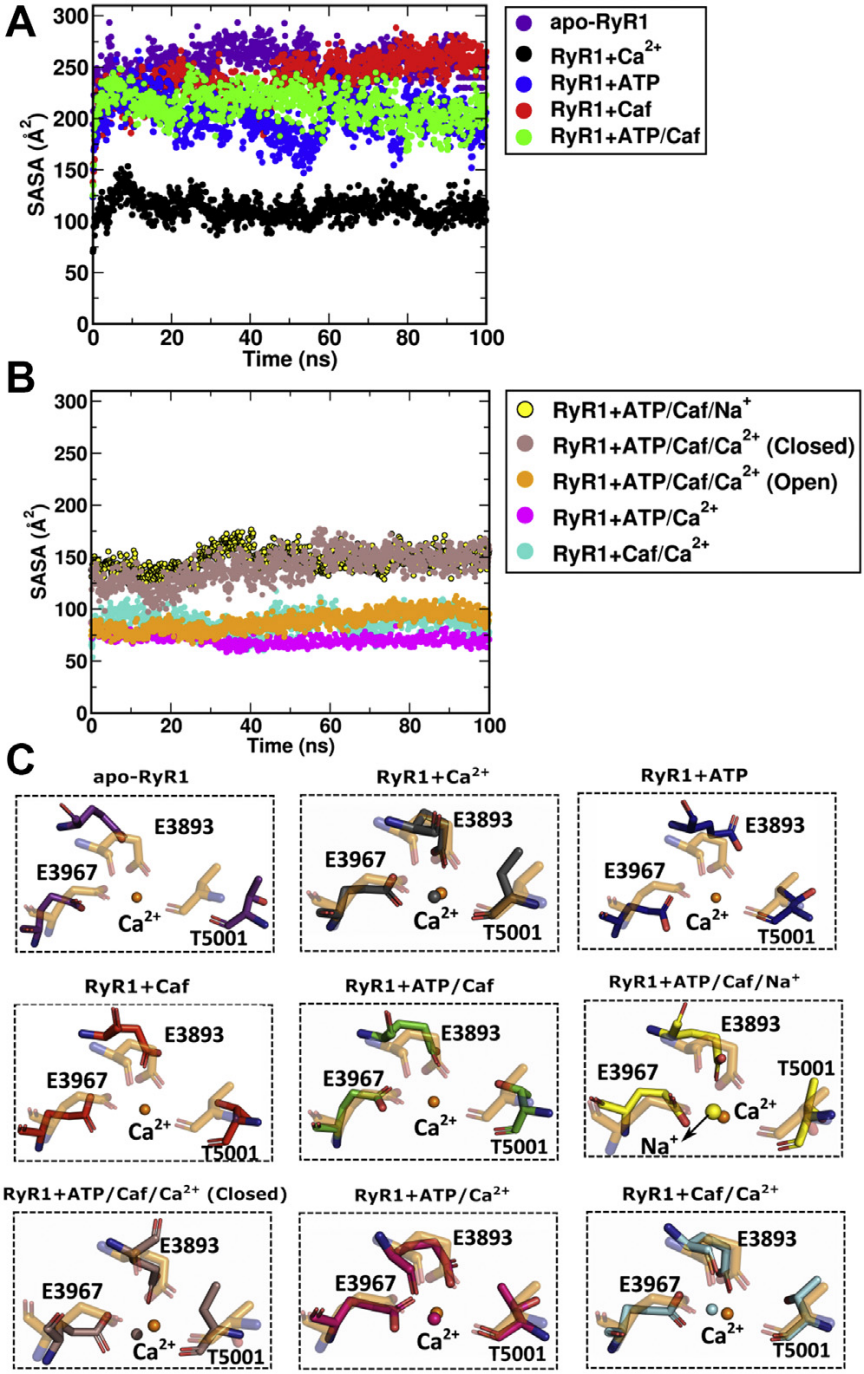
**Quantification of size and dynamics of the Ca**^**2+**^**-binding site in different RyR1 functional states.** To estimate the size of Ca^2+^-binding site, SASA of Ca^2+^-binding site residues Glu-3893, Glu-3967, and Thr-5001 was measured in different RyR1 systems. Evaluation of SASA with respect to the simulation time suggests that the Ca^2+^-binding site exhibits wide and expanded conformation of SASA in the absence of Ca^2+^ (plot *A*). The binding of Ca^2+^ along with ATP, caffeine, or both displayed stable and closed conformation of Ca^2+^-binding site of SASA (plot *B*). *C*, structural superposition of average Ca^2+^-binding sites extracted from last 20 ns of respective simulation trajectories to the average conformation of Ca^2+^-binding site of open RyR1 + ATP/Caf/Ca^2+^ (*orange color*). The Ca^2+^-binding site residues are shown in *stick representation*, and bound ions (Ca^2+^/Na^+^) are shown as *spheres*. The color code for Ca^2+^-binding site is as follows: apo-RyR1 (*purple*), RyR1 + Ca^2+^ (*black*), RyR1 + ATP (*blue*), RyR1 + Caf (*red*), RyR1 + ATP/Caf (*green*), RyR1 + ATP/Caf/Na^+^ (*yellow*), closed RyR1 + ATP/Caf/Ca^2+^ (*brown*), open RyR1 + ATP/Caf/Ca^2+^ (*orange*), RyR1 + ATP/Ca^2+^ (*magenta*), and RyR1 + Caf/Ca^2+^ (*cyan*). To avoid complexity, we maintained same color for Ca^2+^-binding site and corresponding bound Ca^2+^/Na^+^ ions. RyR1, ryanodine receptor type 1; SASA, solvent-accessible surface area.

The homotetrameric RyR1 has four Ca^2+^-binding sites, one in each monomer, at the interface of CSol and CTD. The size of the Ca^2+^-binding sites is inversely proportional to the strength of Ca^2+^ interactions with pocket lining residues. In other words, a wider Ca^2+^-binding site appears to either weaken or inhibit polar interactions of Ca^2+^ with its lining residues. Hence, we quantified the solvent-accessible surface area (SASA) of Ca^2+^-binding site residues Glu-3893, Glu-3967, and Thr-5001 in different RyR1 systems (Fig. 4*A*). Evaluation of SASA with respect to simulation time suggested that the Ca^2+^-binding site is dynamic and unstable in the absence of Ca^2+^. The SASA of Ca^2+^-binding site fluctuated between 197 and 245 Å^2^ in closed apo-RyR1, RyR1 + ATP/Caf, RyR1 + ATP, and RyR1 + Caf states (Table 1). In the presence of Ca^2+^, the Ca^2+^-binding site exhibited a stable conformation in RyR1 + Ca^2+^, RyR1 + Caf/Ca^2+^, RyR1 + ATP/Ca^2+^, and RyR1 + ATP/Caf/Ca^2+^ functional states (Fig. 4*B*). The SASA of Ca^2+^-binding site in Ca^2+^-bound conformations altered between 71 and 121 Å^2^ and exhibited a stable trend throughout respective trajectories (Table 1).

To better understand the alterations in Ca^2+^-binding site conformations in different RyR1 functional states, we extracted the average structures of Ca^2+^-binding sites from the last 20 ns of the respective trajectories and aligned to the Ca^2+^-binding site of RyR1 + Ca^2+^/ATP/Caf (open) system (Fig. 4*C*). The structural alignment of Ca^2+^-binding site of apo-RyR1 and RyR1 + ATP/Caf indicated that the addition of ATP/Caf affected the conformation of the Ca^2+^-binding site in the absence of Ca^2+^. A more compact Ca^2+^-binding site, as observed in open RyR1 + ATP/Caf + Ca^2+^ system, depicts strong polar interactions of Ca^2+^ with the side chains of Glu-3893 and Glu-3967 and the backbone of Thr-5001. However, in closed RyR1 + ATP/Caf/Ca^2+^, Ca^2+^ lost interactions with the CTD residue Thr-5001 and enlarged Ca^2+^-binding site. Altogether, the presence of either ATP or Caf or both significantly modulate the conformation and dynamics of the Ca^2+^-binding site.

Interestingly, the replacement of 0.5 M choline Cl with 0.5 M NaCl increased the stability of RyR1 + ATP/Caf (RMSD = 10.4 *versus* 2.4 Å) and reduced the SASA of the Ca^2+^-binding site from 200 to 147 Å^2^ (Table 1). However, Na^+^ did not affect the RMSD or SASA of the Ca^2+^-binding site in apo-RyR1 (Table 1). This suggested that ATP and Caf binding modulated Ca^2+^-binding site to stabilize Na^+^ binding and interactions. Overlays of Ca^2+^-binding site of apo-RyR1 and RyR1 + RyR1 + ATP/Caf/Na^+^ suggested that Na^+^ strongly interacted with Ca^2+^-binding site residues Glu-3893 and Glu-3957 (Fig. S5). However, we did not observe any interactions between Na^+^ and Thr-5001 in RyR1 + ATP/Caf/Na^+^, which imply that Na^+^ minimally affected the RMSD or SASA of the Ca^2+^-binding site.

**Figure 5:**
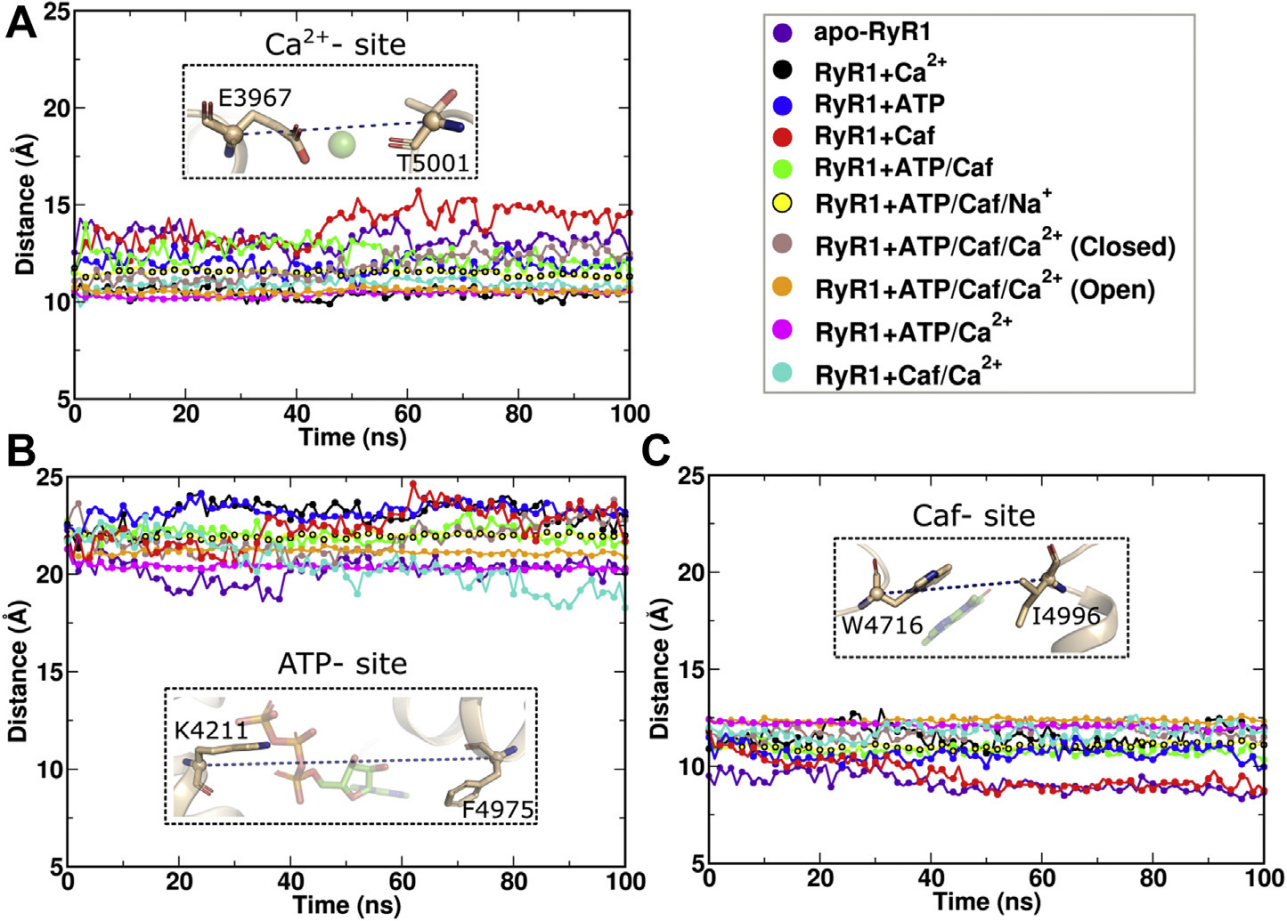
**Quantification of ligand-binding site dimensions to evaluate the dynamics of Ca**^**2+**^**-, ATP-, and caffeine (Caf)-binding sites.** Distance variations between backbone atoms of amino acid residues present on the opposite sides of ligand-binding sites with respect to simulation time. We considered the backbone Cα atoms of (*A*) Glu-3967 and Thr-5001, (*B*) Lys-4211 and Phe-4975, and (*C*) Trp-4716 and Ile-4996 to evaluate the dynamics of Ca^2+^-, ATP-, and Caf-binding sites, respectively. The comparative analysis shows that Ca^2+^-binding site is dynamic and unstable in the absence of Ca^2+^. The ATP-binding pocket is constricted in apo-RyR1, RyR1 + ATP/Ca^2+^, and RyR1 + Caf/Ca^2+^ systems possibly because of structural rearrangements in TaF and CTD. The Caf-binding pocket is constricted and flexible in apo-RyR1 and RyR1 + Caf systems. The residues used for the distance measurement are shown as *sticks* in the insets. ATP and Caf are shown in *stick representation*, and Ca^2+^ is shown in sphere representation. CTD, C-terminal domain; RyR1, ryanodine receptor type 1.

To further capture the conformational changes in Ca^2+^-binding site in different RyR1 functional states, the distance between the backbone Cα atoms of Glu-3967 and Thr-5001 of the Ca^2+^-binding site (d_Ca_^2+^) was measured (Fig. 5*A*). The average and standard deviation of d_Ca_^2+^ is shown in Table S2. Evaluation of the distances with respect to the simulation time suggested that the Ca^2+^-binding site is dynamic and unstable in the absence of Ca^2+^. The distances between Ca^2+^-binding sites Glu-3967 and Thr-5001 (d_Ca_^2+^) fluctuated between 11.9 and 13.9 Å in apo-RyR1, RyR1 + ATP/Caf, RyR1 + ATP, and RyR1 + Caf systems. In the presence of Ca^2+^, d_Ca_^2+^ altered between 10.4 and 11.8 Å and exhibited reduced fluctuations in RyR1 + Ca^2+^, RyR1 + Caf/Ca^2+^, RyR1 + ATP/Ca^2+^, and RyR1 + ATP/Caf/Ca^2+^ (open) systems (Fig. 5*A* and Table S2).

### ATP-binding site

The ATP-binding site is located at the interface of the CTD, TaF, and S6c domains (Fig. 1). The triphosphate tail of ATP is positioned adjacent to the S6c–TaF junction and interacts with positive charged residues Lys-4211, Lys-4214, and Arg-4215 of the TaF domain in all the functional states (Fig. 6). In contrast, significant changes in the interaction of the head group (adenine base) of ATP with the lining residues were predicted. In RyR1 open channels (RyR1 + ATP/Caf/Ca^2+^and RyR1 + ATP/Ca^2+^), the nucleotide head group (adenine base) of ATP is buried deep inside the hydrophobic cleft constituted by hydrophobic residues Met-4954, Phe-4959, Thr-4979, and Leu-4985. These strong hydrophobic interactions may impart stability to the open ATP conformations. The MD trajectories captured additional interactions of triphosphate tail with either Arg-4320 or Arg-4321 or both. However, these interactions were not seen in the absence of Ca^2+^ and Caf binding, which might be due to resultant local structural rearrangements in the activation core.

To capture the conformational changes in ATP-binding site in different RyR1 functional states, we measured distance variations between the pocket lining residues with maximum separation. We chose Lys-4211 from the TaF domain and Phe-4975 from the CTD as ATP pocket lining residues, measured and compared the distance between the backbone Cα atoms (d_ATP_) throughout the simulation trajectories of respective RyR1 functional states (Fig. 5*B* and Table S2). The comparative analysis shows constricted ATP-binding site in apo-RyR1, RyR1 + ATP/Ca^2+^, and RyR1 + Caf/Ca^2+^ systems possibly because of structural rearrangements in the TaF and CTD domains. Surprisingly, the ATP-binding site in RyR1 + ATP/Caf/Ca^2+^ (open) and RyR1 + ATP/Caf/Na^+^ systems depicts less fluctuations and identical trends, which suggest the stabilization of the ATP-binding site.

### Caf-binding site

**Figure 7:**
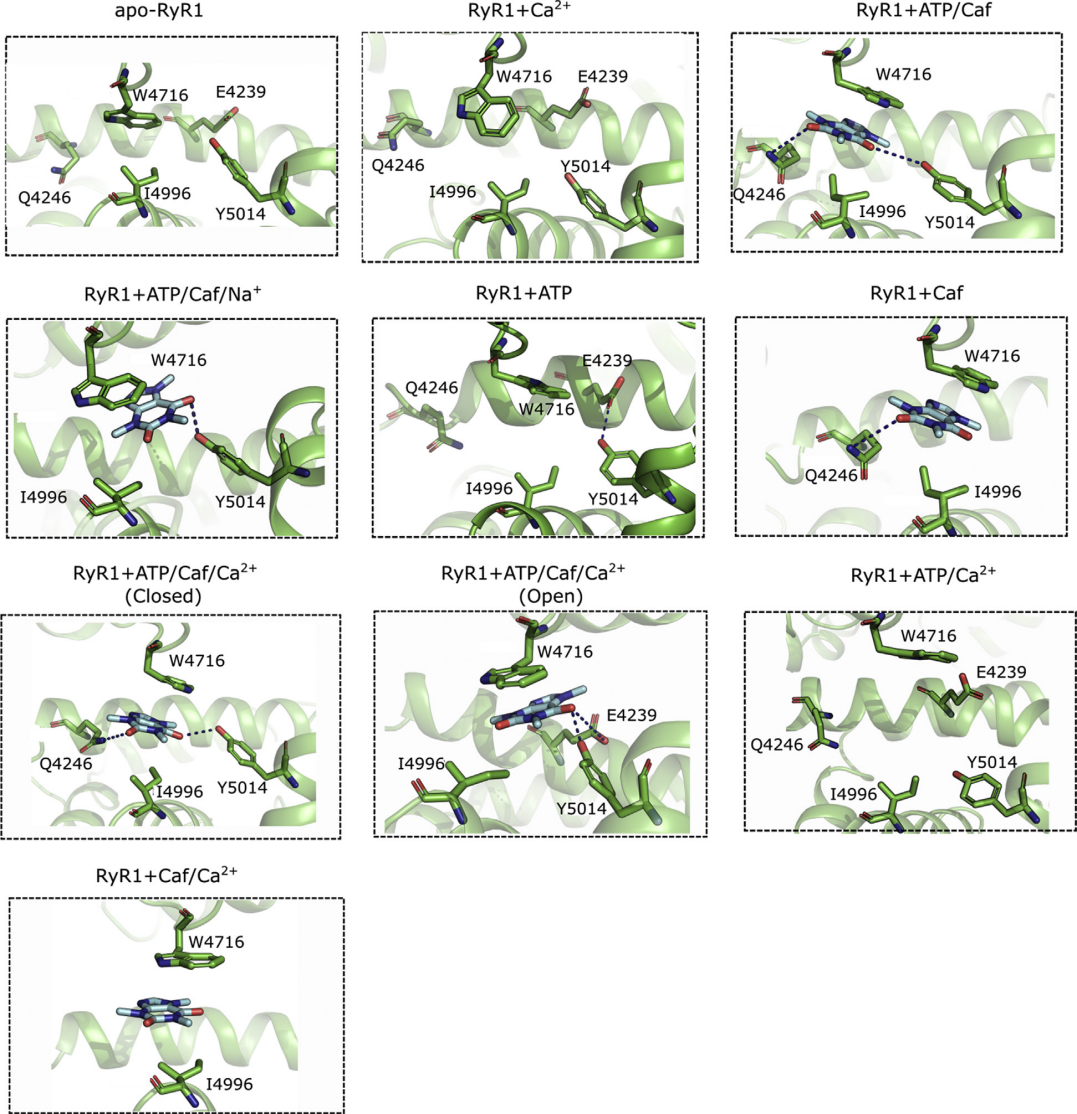
**Comparison of caffeine (Caf)-binding site interactions in different RyR1 functional states.** Interactions of Caf molecule with pocket lining amino acid residues in different RyR1 functional states were estimated by extracting average structures from last 20 ns of respective trajectories. The interaction analysis suggests that Caf molecule induces stacking interactions with Trp-4716 and polar interactions with TaF domain and CTD residues. However, the side chain of Trp-4716 reoriented and blocked Caf-binding pocket in apo-RyR1 and RyR1 + Ca^2+^ systems because of the possible tryptophan-mediated regulation of Ca^2+^ sensitivity. The protein is shown in *green cartoon representation* and Caf-binding site residues in *stick representation*. Caf molecule is rendered in *cyan stick representation*, and H-bond interactions are shown as *blue dotted lines*. CTD, C-terminal domain; RyR1, ryanodine receptor type 1.

The cryo-EM data suggest that the Caf-binding site is located at the interface of the CTD and S2S3 domains (Fig. 7). To understand the effect of ATP and Ca^2+^ on the Caf-binding site, we extracted the average structures from last 20 ns of RyR1 trajectories and compared Caf-site interactions. The binding of either ATP/Caf or Ca^2+^/ATP/Caf reorganized Caf-binding site and induced stacking interactions between purine ring of Caf and the indole group of Trp-4716 from S2S3 domain. The comparative analyses suggest that one of the two carbonyls of Caf formed a H bond with the carboxyl side chain of Glu-4239 and the hydroxyl group of Tyr-5014. The absence of either Ca^2+^ or Caf or Ca^2+^/Caf destabilized the Caf-binding site and modulated its interactions. Specifically, consistent interactions between Caf and the CTD residue: Tyr-5014 was observed in the presence of ATP or ATP/Ca^2+^ or ATP/Na^+^. Furthermore, the interactions of Glu-4239 were absent in all RyR1 closed states irrespective of the presence or the absence of ATP and Ca^2+^. In addition, the reorientation of Trp-4716 side chain that constrained the Caf-binding pocket in the absence of Ca^2+^/ATP/Caf or ATP/Caf reiterates the allosteric interactions between Ca^2+^-, ATP-, and Caf-binding sites to regulate the RyR1 activity.

We further quantified conformational changes in the Caf-binding site in different RyR1 functional states by measuring the distance between the backbone Cα atoms of pocket lining residues. We chose Trp-4716 from S2S3 domain and Ile-4996 from CTD as extreme Caf pocket lining residues and evaluated the distance between their Cα atoms (d_Caf_) with respect to the simulation time. Comparison of d_Caf_ values among the RyR1 variants showed that the Caf-binding pocket is constricted and flexible in apo-RyR1 and RyR1 + Caf systems with d_Caf_ in the range 9.1 to 9.6 Å (Table S2), which might be due to the absence of ATP and Ca^2+^ and the resultant structural changes that bring S2S3 and CTD domains to close proximity. However, the Caf-binding pocket exhibited a stable conformation in the presence of ATP/Caf/Ca^2+^ or ATP/Caf/Na^+^ (Fig. 5*C*). It was surprising to see the extended Caf-pocket conformation in RyR1 + ATP/Ca^2+^ system, even in the absence of Caf, which may be attributed to the concerted movements in the activation core to retain the open state. The low fluctuations of Caf-binding site in RyR1 + ATP/Caf/Na^+^ and RyR1 + ATP/Caf + Ca^2+^ (open) systems suggest the influence of occupied Ca^2+^ pocket on Caf-binding site conformation and possible allosteric interactions between Ca^2+^- and Caf-binding pockets.

## Discussion

Experimental and MD simulations are presented that provide novel insights into the function and associated structural changes of the recombinant skeletal muscle RyR1. Regulation of RyR1 was determined by the three activating molecules Ca^2+^ and AMPPCP/Caf in 0.5 M choline Cl medium using a [^3^H]ryanodine ligand method. The results suggested that Ca^2+^, AMPPCP, or Caf activated RyR1, with Ca^2+^ alone activating RyR1 more efficiently than AMPPCP or Caf alone. In the presence of the three ligands, a concerted activation of RyR1 involved the activator-binding sites. Inhibition of RyR1 by Na^+^ in the absence of Ca^2+^ without eliminating activation by ATP and Caf suggested a specific interaction of Na^+^ with the Ca^2+^-binding site. Computational analysis of cryo-EM densities predicted that the presence of Ca^2+^, ATP, and Caf increased RyR1 protein stability and reduced the SASA and atomic fluctuations of the Ca^2+^-binding site. In the presence of ATP and Caf, Na^+^ was predicted to inhibit RyR1 by binding to the Ca^2+^-binding site in the absence of Ca^2+^ and increase RyR1 stability, which could be due to the stabilization effect of Na^+^ on the RyR1 structure. Comparison of the interactions of the ligand-binding sites, associated structural changes, and dynamics of the pore region in the presence or absence of activators suggested that RyR1 was regulated through an interconnected network of interactions among the Ca^2+^-, ATP-, and Caf-binding sites.

Membrane isolates and purified RyR1s have been extensively used to study RyR1 function and structure often under nonphysiological conditions such as in 0.25 M KCl in [^3^H]ryanodine-binding experiments or 0.5 M NaCl in cryo-EM studies. However, RyR1 activities and structures are affected by the ionic composition of the assay media and other extraneous factors such as lipid composition or the detergent used in purifying and analyzing single specimen (2, 19, 20). Single-channel measurements showed that Ca^2+^ and ATP/Caf partially activated RyR1, and the presence of the three activators increased channel open probability (11). In contrast, analysis of cryo-EM micrographs yielded conformations with closed pore only (apo-RyR1, RyR1+Ca^2+^, and RyR1 + ATP/Caf) or an equal proportion of closed and open channels (RyR1 + ATP/Caf/Ca^2+^). One possible explanation might be that in the presence of Ca^2+^ or ATP/Caf, cryo-EM data scored fewer open channels compared with closed channels. Our MD simulations also indicated that RyR1 maintained a closed pore structure for the apo-RyR1- and Ca^2+^-activated states in choline Cl media. The distinctive pore conformations exhibited by RyR1 + ATP/Ca^2+^ and RyR1 + Caf/Ca^2+^ might be due to relatively weak interactions of Caf with its pocket lining residues compared with the strong electrostatic interactions of the triphosphate tail of ATP with RyR1 and strong hydrophobic interactions of the adenine group with CTD residues. Previous single-channel measurements showed that Ca^2+^ and ATP activated RyR1 by increasing the number and duration of open channel events (21) and Caf by increasing the number and duration of the Ca^2+^/ATP activated RyR1 (22).

Using a [^3^H]ryanodine-binding assay and choline media, we reported previously that RyR1 activities were inhibited by competitive binding of monovalent cations to high-affinity Ca^2+^ activation sites (17). des Georges *et al.* (11) resolved the structures of Ca^2+^-, ATP-, and Caf-binding sites analyzing cryo-EM micrograph densities of the purified RyR1 present in 0.5 M NaCl media, raising the possibility of Na^+^ occupation of the Ca^2+^-binding site in apo-RyR1 and RyR1 + ATP/Caf. However, Na^+^ occupancy was not described. In the present study, we confirmed that replacement of 0.5 M choline Cl with 0.5 M NaCl inhibited [^3^H]ryanodine binding at low micromolar and submicromolar Ca^2+^. Furthermore, computational analysis indicated that placement of Na^+^ in the Ca^2+^-binding site increased the stability of RyR1 + ATP/Caf and the absence of Ca^2+^ decreased the SASA of the Ca^2+^-binding site to a size more comparable to that of the RyR1 + ATP/Caf determined in cryo-EM studies (200 Å^2^ without Na^+^ and 147 Å^2^ with Na^+^ *versus* 133 Å^2^ in cryo-EM; Table 1). Na^+^ was not stably bound to the Ca^2+^-binding site and did not affect RMSD or SASA of the Ca^2+^-binding site of the apo-RyR1. This suggests that ATP and Caf binding modulated Ca^2+^-binding site conformation that stabilized Na^+^ binding to RyR1 residues Glu-3893 and Glu-3967. However, it remains unclear why Na^+^ did not occupy the Ca^2+^-binding site of the native detergent-purified RyR1. Nonetheless, in the present study, the Ca^2+^-binding site of the membrane-bound recombinant RyR1 could bind Na^+^ and affect RyR1 stability and function.

Structural analysis has indicated that the binding of ATP, Caf, and Ca^2+^ produces global changes affecting the extension of RyR1 and RyR2 out of the SR membrane (11, 12). Time-resolved movies obtained from single-particle cryo-EM snapshots of RyR1 revealed that conformational changes in the activation domain and specific to the N-thermal domains facilitate the formation of Ca^2+^-binding site conformations required to overcome the high energy required for Ca^2+^ binding (23). The results also indicated that activated states can be present in the apo-RyR1 state at low probability. Experimental evidence for the involvement of the large cytoplasmic shell in modifying RyR1 and RyR2 activities include the presence of disease-associated mutants (4, 6, 24, 25); location of binding sites for regulatory proteins, such as FK506-binding protein and calmodulin; and post-translational reactions involving RyR1 phosphorylation and redox modifications (2). A more flexible conformation on phosphorylation of RyR2 has been suggested to facilitate the transition to the open state, whereas the binding of FK506-binding protein enhanced rigidity and stabilized the RyR2 closed state (26). MH-associated RyR1-R615C (27) and RyR1-R164C mutants (25) increased channel openings by affecting interaction between regions in the cytoplasmic shell accompanied by alterations of the Ca^2+^-binding site (25). Furthermore, the disease-associated mutations can induce conformational changes to facilitate Ca^2+^ binding and increase channel open probability (28). In support, our MD simulations predicted that under physiological conditions, that resembled the resting state of skeletal muscle, the RyR1 + ATP system maintained flexible conformation with a high RMSD ∼10 Å. However, the presence of Ca^2+^ and ATP in RyR1 + ATP/Ca^2+^ has drastically reduced the RMSD to ∼1.4 Å and stabilized the RyR1 conformation (Fig. S3 and Table 1).

The Cryo-EM data suggested that the binding sites of Ca^2+^, ATP, and Caf are located at the interfaces of the CTD, TaF, S2S3, and S6c domains. The Ca^2+^-binding site is formed by side chains of Glu-3893 and Glu-3967 in CSol domain, and the backbone carbonyl of Thr-5001 in CTD. Loss of Ca^2+^-dependent activation of RyR1-Glu-3893Q and RyR1-Glu-3967Q variants and reduced apparent binding affinity for Ca^2+^ by RyR1-Thr-5001A indicated the significance of Glu-3893, Glu-3967, and Thr-5001 residues in forming a functional Ca^2+^-binding site (29). MD simulations suggested that the binding of Ca^2+^, ATP, and Caf to their respective binding sites rearranges CSol, CTD, S2S3, and TaF domains and promote channel pore dilation. Specifically, MD simulations in the absence of Caf provided additional direct insights into the allostery mediated by the two physiological activators—Ca^2+^ and ATP. Comparison of RyR1 dynamics between RyR1 + ATP/Caf/Ca^2+^ (open) and RyR1 + ATP/Ca^2+^ indicated that RyR1 + ATP/Ca^2+^ displayed a more stable open protein structure (RMSD = 1.4 *versus* 2.5 Å) and smaller Ca^2+^-binding site (SASA = 70.7 *versus* 110 Å^2^). Furthermore, while maintaining unstable protein conformation with RMSD ∼10 Å, the RyR1 + Caf/Ca^2+^ also exhibited constricted Ca^2+^-binding site with SASA of 86.9 Å^2^ in comparison to the Ca^2+^-binding site of RyR1 + Ca^2+^ (SASA = 121 Å^2^). Together, the results suggest that the ATP and Caf-binding sites affect the stability and structure of the Ca^2+^-binding site through allosteric regulation.

The ATP-binding site is situated at the interface of the S6c-CTD-TaF domains with the trisphosphate tail binding to the TaF domain positively charged residues Lys-4211, Lys-4214, and Arg-4215. The comparison of ATP interactions among RyR1 (PDB IDs: 5TAL and 6M2W) (11, 30) and RyR2 (PDB: 6JIY) (31) structures suggest that ATP binding is identical and interacting residues are well conserved in RyR1 and RyR2 (Fig. S6 and Table S3). However, subtle differences exist in ATP orientation and interactions. For instance, the triphosphate tail of ATP interacts with Lys4211 and Arg4215 side chains in 5TAL (11); Lys4214 side chain in 6M2W (30); and Lys4170 (Lys4214 in RyR1); and Arg4171 (Arg4215 in RyR1) in 6JIY (RyR2) (31). Furthermore, the adenine base interacts with C4958 backbone in 5TAL; H4983 backbone in 6M2W (30); and L4916 backbone (L4985 is the corresponding residue in RyR1) in 6JIY (RyR2) (31). Despite these minor differences in polar interactions, the pocket lining residues of ATP are identical between RyR1 and RyR2 conformations.

Our MD trajectories captured additional interaction of the triphosphate tail with either Arg-4320 and/or Arg-4321, which were not seen in closed ATP-occupied conformation (RyR1 + ATP). Significant structural changes were predicted for the interaction of the adenine group with CTD residues in the open and closed RyR1 conformations. In the closed RyR1 + ATP, RyR1 + ATP/Caf, and RyR1 + ATP/Caf/Ca^2+^ conformations, the adenine head group formed polar interactions with CTD residues: His-4983, Leu-4980, and Glu-4955, respectively. Lindsay *et al.* (18) group showed that the trisphosphate tail of ATP can alone activate RyR2 by interacting with the TaF domain. Furthermore, the presence of adenosine along with the triphosphate group regulated dynamics of triphosphate moiety and reproduced the effects of ATP (18). Consistent with these observations, our data depicted that the open states of RyR1 + ATP/Ca^2+^ and RyR1 + ATP/Caf/Ca^2+^ systems were attributed to polar interactions of ATP's trisphosphate tail with the TaF (Fig. 6). Furthermore, in RyR1 open states, the adenine group was buried inside the hydrophobic cleft formed by CTD residues Met-4954, Phe-4959, Thr-4979, and Leu-4985. Strong hydrophobic interactions of adenosine head group with the CTD residues stabilized ATP in an extended conformation. Addition of Ca^2+^ to the closed RyR1 + ATP and RyR1 + ATP/Caf states led to channel opening. Hence, we can hypothesize that the triphosphate moiety of ATP is important for structural changes associated with the RyR1 channel gating and activation. In the future, we will design point mutations in TaF (trisphosphate-binding interface) and/or CTD (adenosine-binding interface) to probe the mechanism of RyR1 activation by ATP and its analogs.

The Caf-binding site is formed by TaF residue Glu-4239, S2–S3 residue Trp-4716, and CTD residues Ile-4996 and Tyr-5014. Previous studies showed that the binding of Caf in the presence of an adenine nucleotide altered the orientation of Trp-4716 in the TaF domain and position of Ile-4996 in the CTD. The induced conformational changes in CTD modified the Ca^2+^-binding pocket structure for more favorable binding of Ca^2+^ (16). In [^3^H]ryanodine binding studies and MD simulations presented here, we compared the conformational changes in the Caf-binding site among different RyR1 states in the absence or presence of either Ca^2+^, or ATP, or both. The absence of either Ca^2+^, or ATP, or Ca^2+^/ATP induced structural changes in the RyR1 activation core and altered the orientation and interactions of Caf. The binding of Ca^2+^/ATP/Caf to the open RyR1 reorganized the Caf-binding site and induced stacking interactions between the purine of Caf and the indole group of Trp-4716 from S2S3 domain, carboxy group of Glu-4239 from TAF domain, and the hydroxyl group of Tyr-5014 from CTD (Fig. 1). Our MD data suggest that the side chain of Trp-4716 reoriented and blocked Caf-binding pocket in apo-RyR1 and RyR1 + Ca^2+^ systems because of the possible tryptophan-mediated regulation of Ca^2+^ sensitivity as predicted by Murayama *et al.* (16). Furthermore, comparison of the Caf-binding site from different RyR1 functional states suggests that the interactions of Tyr-5014 with Caf were persistent in the presence of ATP, which could be due to ATP-induced structural transitions in the CTD. In addition, the Caf-binding pocket exhibited extended conformation in RyR1 + ATP/Ca^2+^ system, even in the absence of Caf, which can be attributed to the concerted movements in the activation core to retain the open state (Fig. 7). Our experimental data complemented by computational findings demonstrate that Ca^2+^- and ATP-binding sites communicated with the Caf-binding site to regulate Caf binding and RyR1 functional activity.

Seminal freeze-fracture EM studies suggested a strong mechanical excitation–contraction (EC) coupling between skeletal muscle calcium channel (Cav_1.1_) and RyR1 receptor (32, 33, 34). The EM studies further demonstrated that Cav_1.1_ interacts with the cytoplasmic region of RyR1, which is isoform specific for both proteins. This EC coupling mechanism between Cav_1.1_ and RyR1 *via* protein–protein and other allosteric interactions, such as with STAC3, activates SR calcium release (35). While skeletal EC coupling does not require increase in cytosolic Ca^2+^ to activate RyR1, Ca^2+^ influx through Cav_1.2_ during an action potential is essential to activate RyR2, that is, calcium-induced calcium release is essential for cardiac muscle contraction. It is worth noting here that the enhanced Caf-induced skeletal muscle contraction is a hallmark for diagnosis for MH (36), a RyR1 mutation–associated myopathy. A number of MH-linked RyR1 mutations also increase activation of the channel by Ca^2+^. Although it remains elusive whether RyR1 activation by Cav_1.1_ or endogenous and exogenous effectors (Ca^2+^, ATP, or Caf) follow similar mechanism, it is important to gain molecular insights in RyR1 activation by effector ligands to understand skeletal muscle pathophysiology. In this aspect, our current study suggested an enhanced RyR1 activity through allosteric interactions among Ca^2+^-, ATP-, and Caf-binding sites. Furthermore, the location of the activator-binding sites with respect to the pore region suggests that the transmission of communications from ATP- and Caf-binding sites to the pore region merges with the Ca^2+^-binding site—pore pathway to enhance channel opening probability.

**Figure 8:**
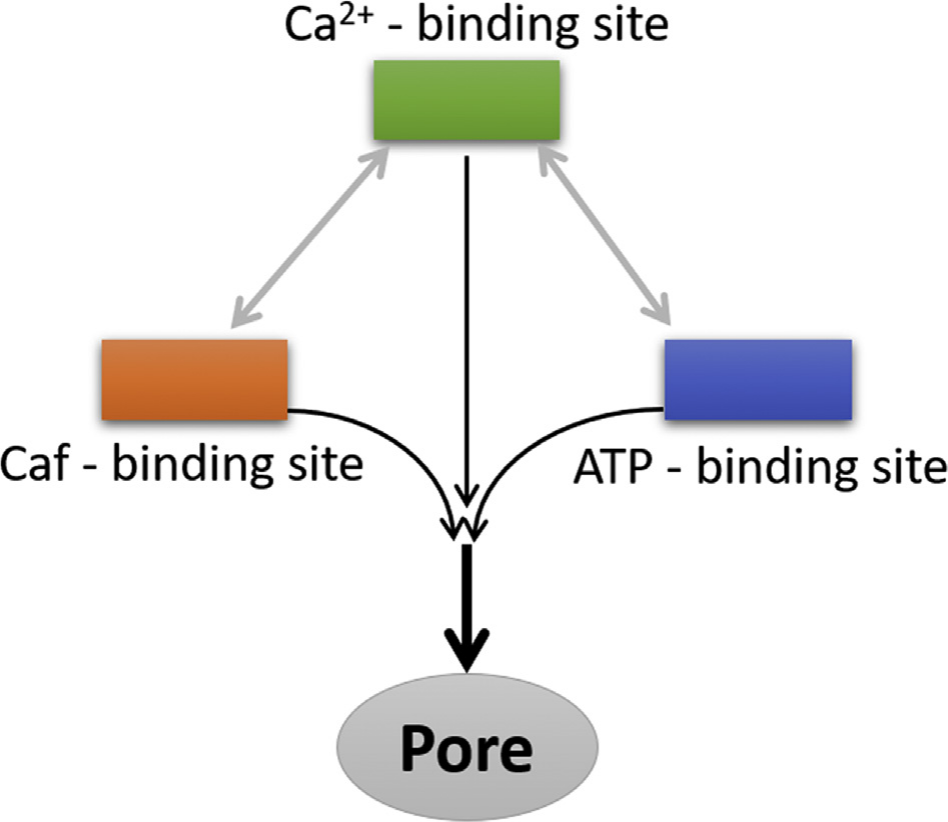
**Schematic representation of allosteric interactions among the ligand sites to enhance RyR1 channel opening probability.** ATP- and caffeine-binding sites communicate with the Ca^2+^-binding site in the absence of Ca^2+^ and merge with the Ca^2+^-binding site—pore pathway in the presence of Ca^2+^ to amplify RyR1 activity. RyR1, ryanodine receptor type 1

To summarize, the allosteric interactions among three ligand-binding sites and the pore region of RyR1 were investigated in different functional states. Our simulations indicate that the rearrangements in the activation core because of the binding of Ca^2+^, ATP, and Caf at their respective binding sites dilate the pore region. An illustrative representation based on the current findings suggests that the transmission of communication from the Ca^2+^-binding site to the pore region is augmented in the presence of ATP and Caf by their respective binding sites in both the open and closed RyR1 structures (Fig. 8). Enhanced RyR1 activity complemented by structural rearrangements of the closed Ca^2+^-binding site in the presence of ATP and Caf suggests that the ATP- and Caf-binding sites could communicate with and modulate the Ca^2+^-binding site in the absence of Ca^2+^. Furthermore, experimental evidence suggests an increased RyR1 channel opening probability in the presence of Ca^2+^, ATP, and Caf. Our computational MD data on RyR1 + ATP/Ca^2+^ and RyR1 + Caf/Ca^2+^ predicted that the presence of ATP was sufficient to increase the channel opening probability. Structural constraints and the location of the activator-binding sites with respect to the pore region suggest that the pathways originated from ATP- and Caf-binding sites to pore region merge with the Ca^2+^-binding site—pore pathway to improve the channel opening probability. Nonetheless, the exact position where the pathways are joined may be revealed using site-directed mutagenesis.

## Experimental procedures

### Materials

[^3^H]Ryanodine was obtained from PerkinElmer Life Sciences; protease inhibitors from Roche; AMPPCP , Caf, and choline Cl from Sigma–Aldrich; jetPRIME transfection reagent from Polyplus; and phospholipids from Avanti Polar Lipids.

### Membrane preparation

RyR1-WT vectors were transiently expressed in human embryonic kidney 293 cells using jetPRIME reagent (Polyplus) according to the manufacturer's instructions. Cells were maintained at 37 °C and 5% CO_2_ in Dulbecco's modified Eagle's medium containing 10% fetal bovine serum and replated the day before transfection (37). RyR1 plasmids were transiently expressed in human embryonic kidney 293 cells using 10 μg plasmid-/10-cm culture dish, grown at 35 °C for 72 h, and washed twice with PBS containing complete protease inhibitor cocktail. Cells were harvested in the same solution by scrapping, collected by centrifugation, and stored in a liquid nitrogen freezer. To prepare membrane fractions, cells were resuspended in 0.25 M choline Cl, 0.3 M sucrose, 20 mM imidazole, pH 7 containing 1 mM EGTA, 1 mM glutathione disulfide, and protease inhibitor cocktail and homogenized using a Tekmar Tissumizer for 5 s, setting 35,000 rpm. Cell homogenates were centrifuged, pellets were washed, and resuspended in 0.25 M choline Cl, 0.3 M sucrose, 20 mM imidazole, pH 7 solution containing protease inhibitor cocktail and stored in a liquid nitrogen freezer.

### [^3^H]Ryanodine binding

Ryanodine binds with high specificity to RyR1 and is widely used to probe RyR activity and content (38). Unless otherwise indicated, the functional effects of Ca^2+^, ATP, and Caf were determined by incubating crude membrane isolates for 20 to 24 h at 24 °C in 0.5 M choline Cl media containing 20 mM imidazole, pH 7.0, and the indicated concentrations of Ca^2+^, AMPPCP (nonhydrolyzable ATP analog), Caf, 2 to 5 nM [^3^H]ryanodine, and protease inhibitors. B_max_ value of [^3^H]ryanodine binding was determined by incubating crude membrane isolates for 20 to 24 h at 24 °C in 0.6 M choline Cl containing 20 mM imidazole, pH 7.0, 0.15 mM Ca^2+^, 20 nM [^3^H]ryanodine, and protease inhibitors. Nonspecific binding was determined in the presence of 10 μM unlabeled ryanodine. Amounts of bound [^3^H]ryanodine were determined using a filtration assay (39).

### Data analysis

Free Ca^2+^ concentrations were determined using a Ca^2+^-selective electrode. Free Ca^2+^ concentrations following the addition of 2 mM ATP were calculated using MaxChelator and constants from the Theo Schoenmakers' Chelator (http://maxchelator.stanford.edu/downloads.html). Differences between samples were analyzed using two-way ANOVA followed by Holm–Sidak method. *p* < 0.05 was considered significant.

### MD simulations

To understand the mechanism of coregulation and synergy between the RyR1 activators (Ca^2+^, ATP, and Caf), we performed MD simulations on cryo-EM structures of RyR1 in different ligand-bound states (Table S1) (11). Briefly, we considered unliganded RyR1 (denoted “apo RyR1,” PDB: 5TB0), Ca^2+^-only bound RyR1 (denoted “RyR1 + Ca^2+^,” PDB: 5T15), ATP- and Caf-bound RyR1 (denoted “RyR1 + ATP/Caf,” PDB: 5TAP), Ca^2+^-, ATP-, and Caf-bound closed RyR1 (denoted “RyR1 + ATP/Caf/Ca^2+^ [closed],” PDB: 5TAQ), and Ca^2+^-, ATP-, and Caf-bound open RyR1 (denoted “RyR1 + ATP/Caf/Ca^2+^ [open],” PDB: 5TAL) as starting structures to simulate the dynamics of RyR1 in various ligand-bound states. Furthermore, these systems will also serve as control systems to understand the ligand site conformations and pore dynamics in the presence or absence of modulators. To mimic the RyR1 + ATP/Caf system in 0.5 M NaCl concentration, we manually docked Na^+^ ions to Ca^2+^-binding sites of RyR1 + ATP/Caf. We placed the Na^+^ ion in such a way that the side chains of Glu-3893, Glu-3967, and Thr-5001 could induce electrostatic interactions with the Na^+^ ion. We referred this system throughout the article as RyR1 + ATP/Caf/Na^+^. To understand the individual role of ATP and Caf on Ca^2+^-binding site, we generated RyR1 + ATP and RyR1 + Caf conformations by removing Caf and ATP molecules, respectively, from RyR1 + ATP/Caf system (PDB: 5TAP). Furthermore, we generated RyR1 + ATP/Ca^2+^ and RyR1 + Caf/Ca^2+^ systems by removing Caf and ATP, respectively, from RyR1 + ATP/Caf/Ca^2+^ (open) system. The details of MD system configurations, experimental conditions, pore status, and abbreviations used for each system are summarized in Table S1.

The long missing segments in cryo-EM structures with residue range: 1875 to 1921, 4254 to 4539, and 4588 to 4625 were replaced by short Gly loops of 8, 17, and 48 residues, respectively, using the SWISS-MODEL tool (https://swissmodel.expasy.org/) (40). Loop optimization was performed using Modeller-9v18 (https://salilab.org/modeller/) (41) to refine modeled loop regions and abolish atomic clashes. However, replacing two transmembrane helices with short Gly loops will influence the dynamics and stability of RyR1, which is a limitation of our computational modeling study. The transmembrane domain regions of modeled structures were embedded in 1-palmitoyl-2-oleoyl-glycero-3-phosphocholine—lipid bilayers of dimensions 217 Å × 217 Å × 37 Å using the CHARMM-GUI tool (42, 43). A snapshot of the membrane-inserted RyR1 system is shown in Figure S7. Protein atoms were restrained using harmonic potential and gradually releasing the restraints in several short-equilibrated simulations to nullify clashes between protein and membrane atoms. CHARMM36 force field (44, 45, 46, 47) was employed on protein, membrane lipids, ATP, and Caf molecules using GROMACS-2020 (https://www.gromacs.org/) (48). The force field parameters of Caf were generated using the CGenFF server (49). The appropriate charge adjustments were made to neutralize penalty scores. The Caf topology was converted to GROMACS format using the *cgenff_charmm2gmx.py* script provided in CHARMM Web site (http://mackerell.umaryland.edu/charmm_ff.shtml#gromacs) (43). The equilibrated membrane-inserted RyR1 structures were placed in a box with dimensions 341.1 Å × 341.1 Å × 272.2 Å and explicitly solvated using the TIP3P water model. The water molecules trapped in the lipid bilayer were removed, and a salt concentration of 0.15 M KCl was maintained by replacing appropriate number of water molecules with K^+^ and Cl^−^ ions. We used KCl instead of choline Cl and ensured that neither Na^+^ nor K^+^ occupied the Ca^2+^-binding site in apo-RyR1 or RyR1 + ATP/Caf systems. The solvated systems were optimized using 50,000 steps of conjugate gradients followed by 50,000 steps of steepest-descent algorithms. Thermodynamic parameters such as temperature and pressure were maintained at 323 K temperature and 1 bar pressure using Parrinello–Rahman barostat (50). The equilibration simulations were performed for 10 ns with a time step of 2 fs. The hydrogen bonds were constrained using the LINCS algorithm (51). Long-range electrostatics were evaluated using particle mesh Ewald with a cutoff of 12 Å (52). The simulations were performed for 100 ns with a time step of 2 fs using GROMACS-2020 package (48). The 3D structural visualization of average RyR1 conformations extracted from respective trajectories was rendered using the PyMOL Molecular Graphics System (53). Detailed description of simulated system configurations is shown in Table S1.

### RMSD

RMSD of protein residues is a common measure to estimate the dynamics of protein structures. The mean square displacement of protein backbone atoms with respect to their initial positions estimates the RMSD of protein residues in a simulation trajectory as shown in the following equation:

$$\mathrm{RMSD}=\sqrt{\frac{1}{N}\sum_{i=1}^{N}d_{i}^{2}}$$

where *d*_*i*_ is the distance between backbone atom *i* and its reference position, and *N* is the total number of atoms.

### SASA

We used the *gmx sasa* tool to estimate the SASA of Ca^2+^-binding site with respect to simulation time (54). Briefly, *gmx sasa* evaluates SASA by rolling and tracing the path of a sphere with *r* = 1.4 Å along the van der Waals surface of Ca^2+^-binding site. The SASA of all four Ca^2+^ sites across respective MD trajectories was averaged and compared for meaningful data extraction.

## Data availability

The data that support the findings of this study are available from the corresponding author, V. R. C., upon reasonable request.

## Supporting information

This article contains supporting information (55).

## Conflict of interest

The authors declare that they have no conflicts of interest with the contents of this article.
