# Supplementary material for: Structural and functional interactions between the Ca2+-, ATP-, and caffeine-binding sites of skeletal muscle ryanodine receptor (RyR1)
Source: J Biol Chem. 2021 Aug 2;297(3):101040. doi: 10.1016/j.jbc.2021.101040 (PMC8408527; doi:10.1016/j.jbc.2021.101040)
Supplement: Tables S1–S7 and Figures S1–S3 [file mmc1.docx]

**Supplemental Information**

**Structural and functional interactions between the Ca^2+^, ATP, and caffeine**

**binding sites of skeletal muscle ryanodine receptor (RyR1)**

Venkat R. Chirasani*, Daniel A. Pasek, and Gerhard Meissner

Department of Biochemistry and Biophysics, Univerity of North Carolina, Chapel Hill,

NC 27599, USA

**Table S1. Ligand site dimensions**

**
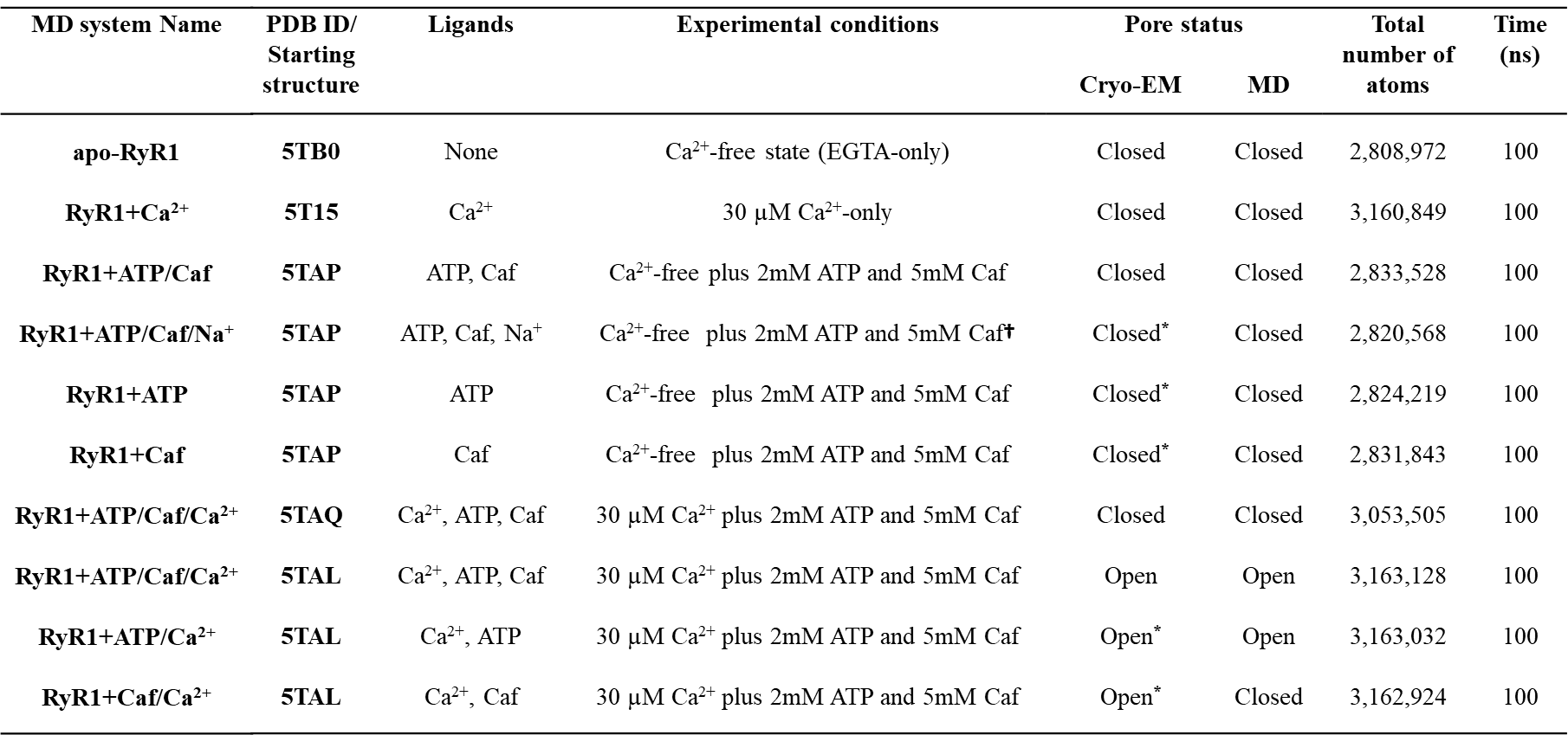
**

† This system has 0.5 M NaCl.

* Pore status of template/base structure.

**Table S2. Quantification of ligand binding site dimensions**


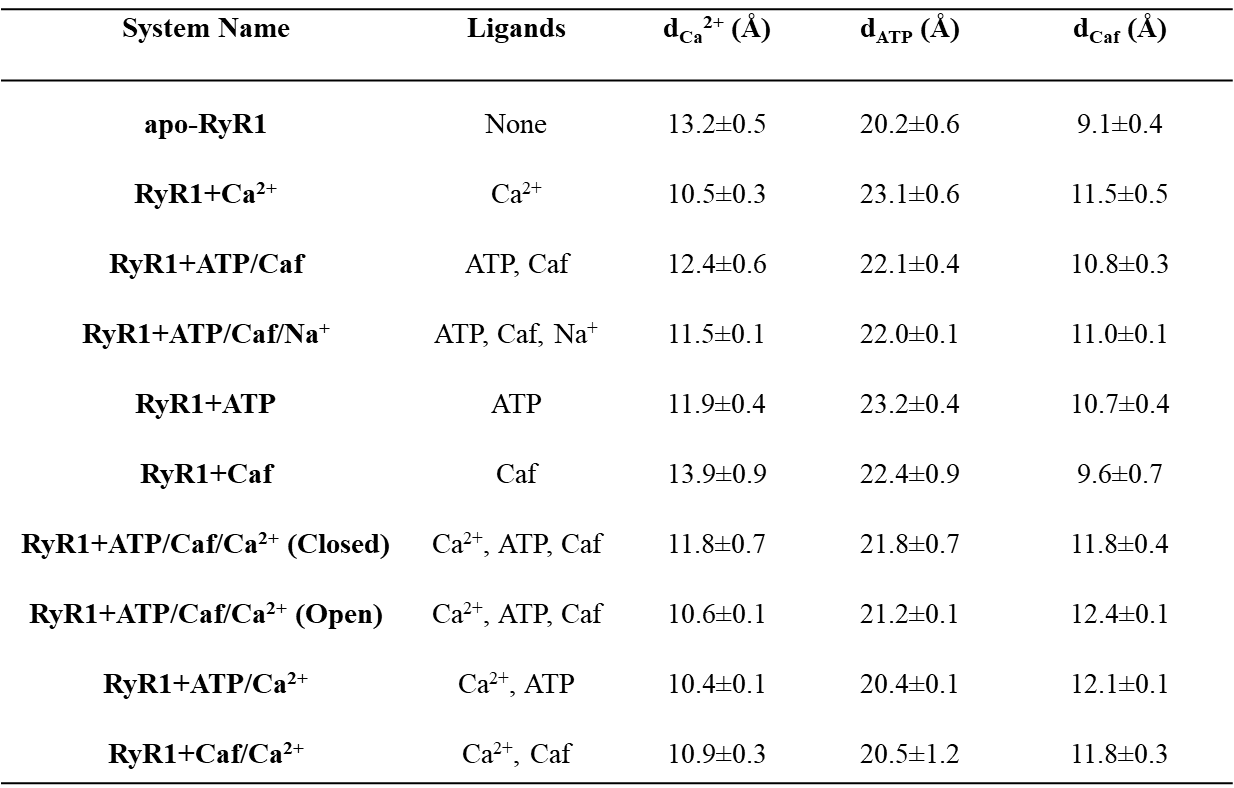


**Table S3. Comparison of ATP-site interactions**


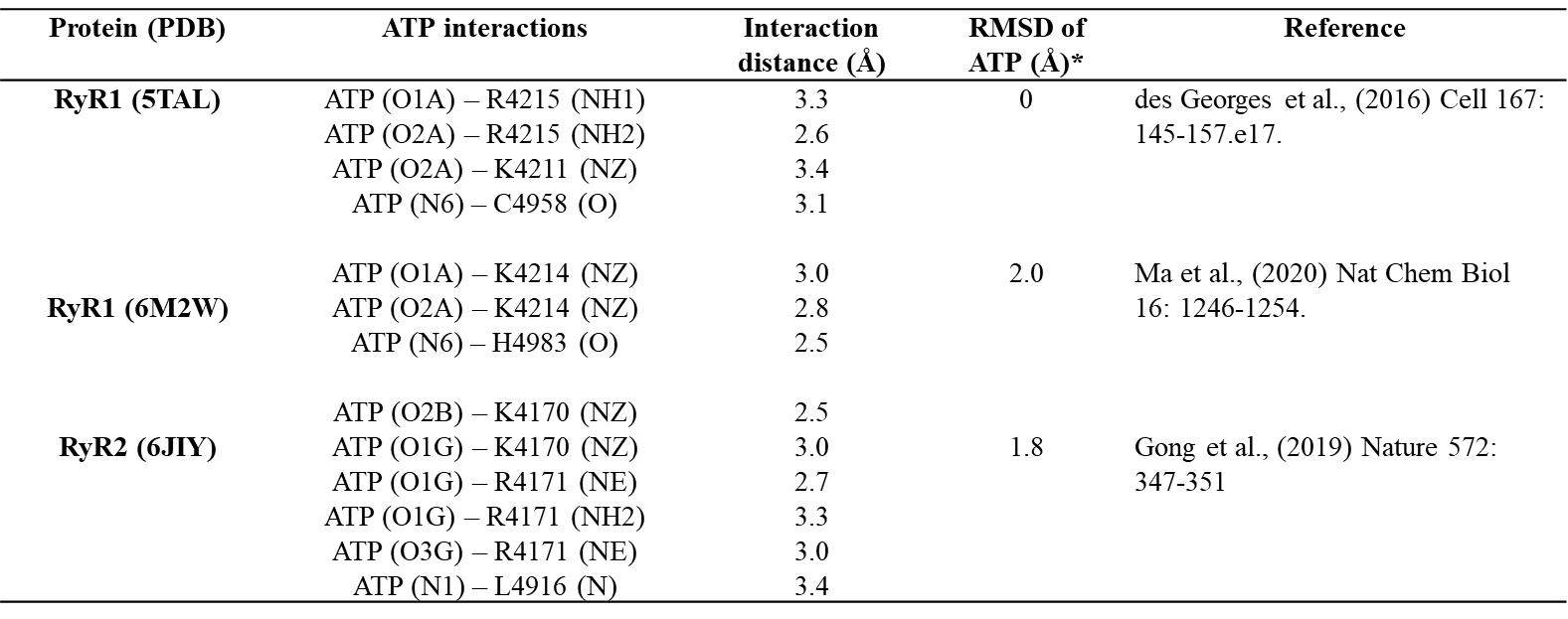


*RMSD calculations were performed with respect to ATP in 5TAL as base structure.


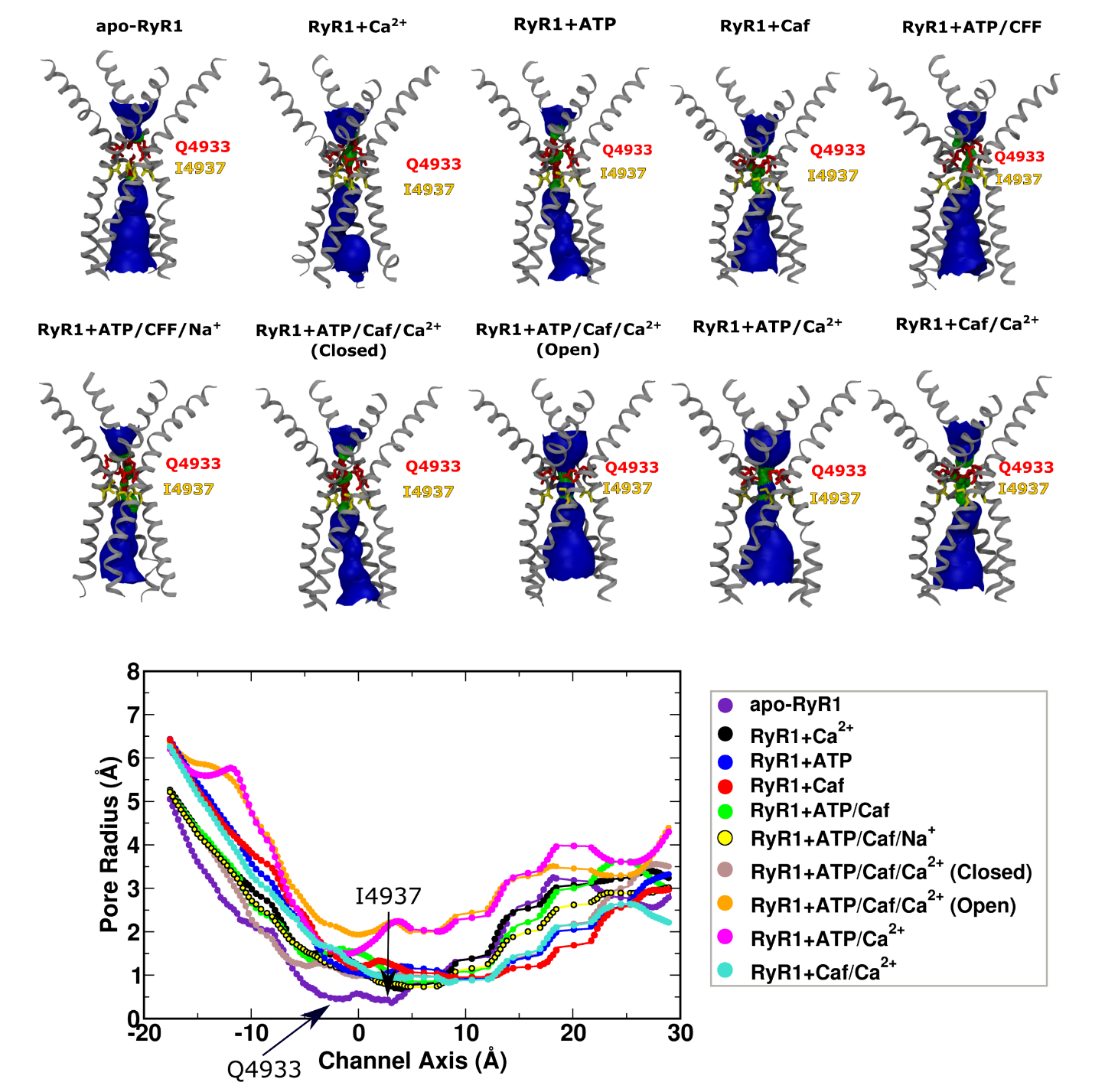


**Fig. S1. Comparison of Ca^2+^ channel pore profiles of RyR1 functional states.** A, Inner solvent accessible surface of pore channel was calculated by HOLE (55) and is shown in blue surface representation with pore helices shown in gray. Channel gating residues Gln-4933 and Ile-4937 are shown in stick representation. B, Evolution of channel pore radius with respect to channel axis. One structure for every ns of the simulation trajectories was sampled and processed using HOLE (55) to compute the pore profiles. Pore constriction site residues Gln-4933 and Ile-4937 are highlighted on channel axis for reference.


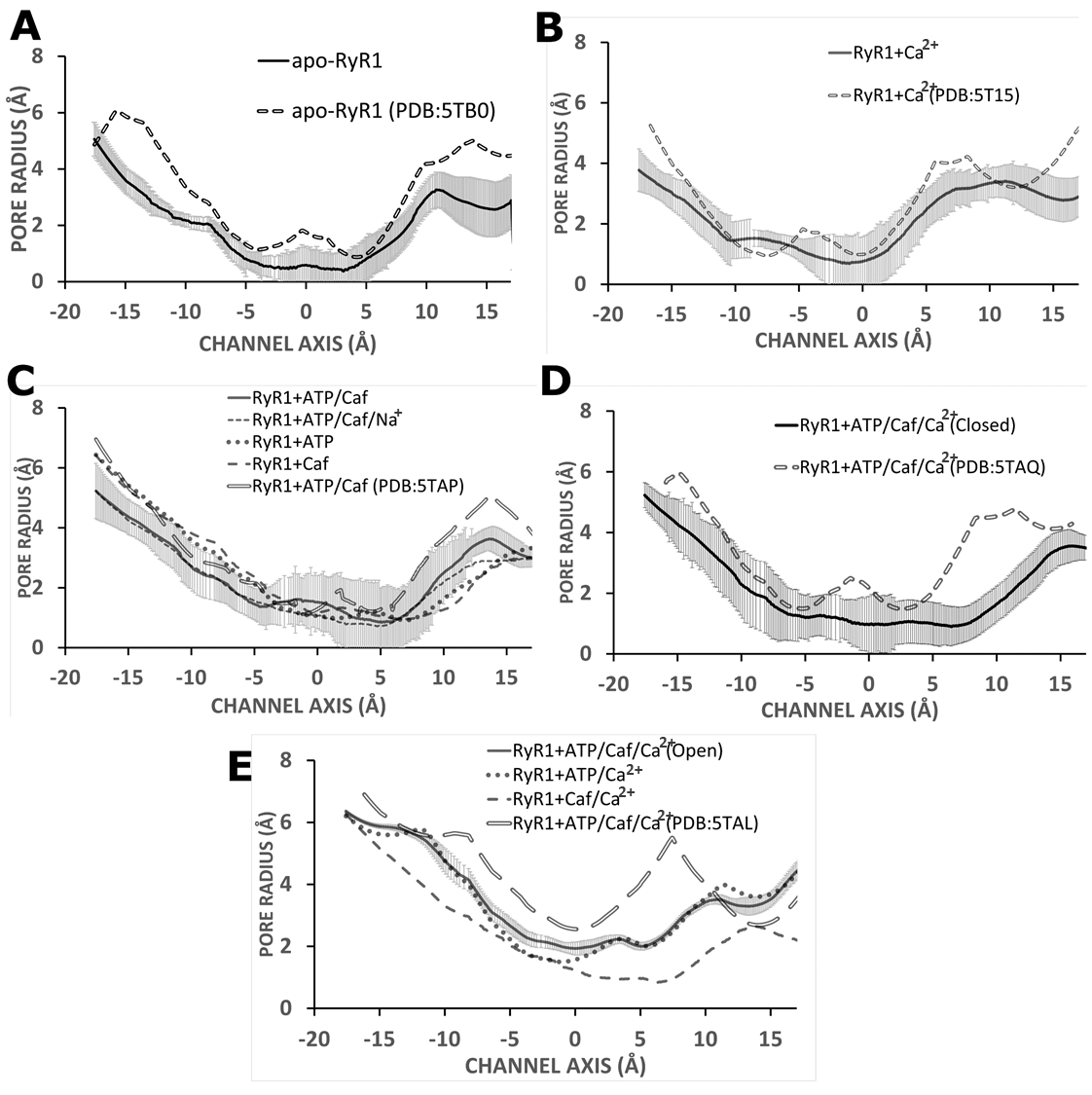


**Fig. S2. Comparison of average pore radius profiles from MD trajectories with respective starting PDB structures.** Since RyR1+ATP/Caf (PDB: 5TAP) was utilized as starting structure for RyR1+ATP/Caf, RyR1+ATP/Caf/Na^+^, RyR1+ATP, and RyR1+Caf simulations, we showed the average pore profiles of these systems in a single panel. Similarly, open RyR1+ATP/Caf/Ca^2+^ (PDB:5TAL) cryo-EM structure was used as starting conformation to initiate MD simulations on RyR1+ATP/Caf/Ca^2+^ (Open), RyR1+ATP/Ca^2+^, and RyR1+Caf/Ca^2+^ systems.


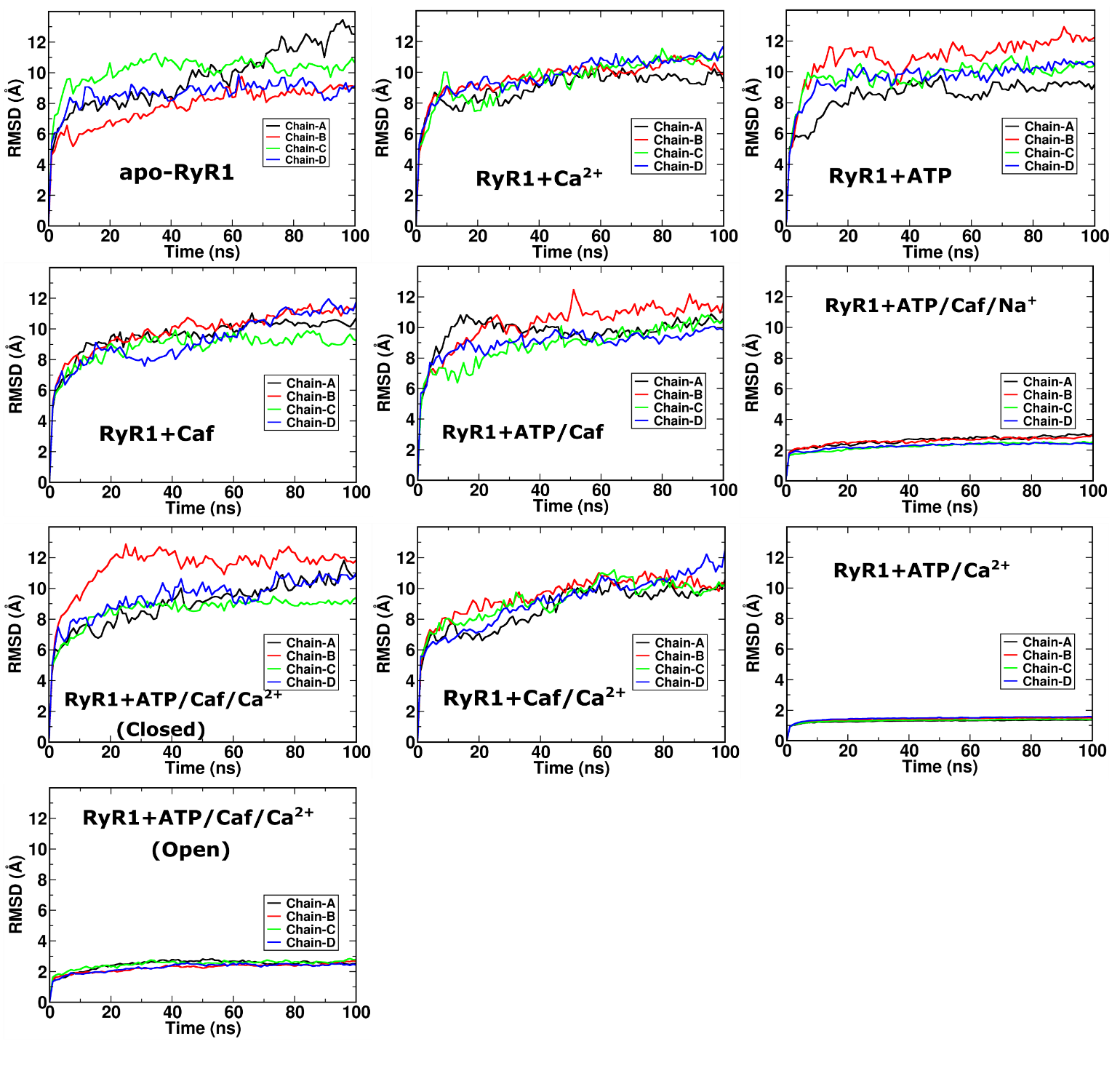


**Fig. S3. Evaluation of protein stability with respect to simulation time in different RyR1 functional states.** The root-mean square deviation (RMSD) of protein backbone atoms with respect to the starting structure estimates the stability of RyR1 protein structure. Comparison of RMSD profiles among the MD systems with RyR1 in different functional states highlights the significance of ligand binding on RyR1 stability.


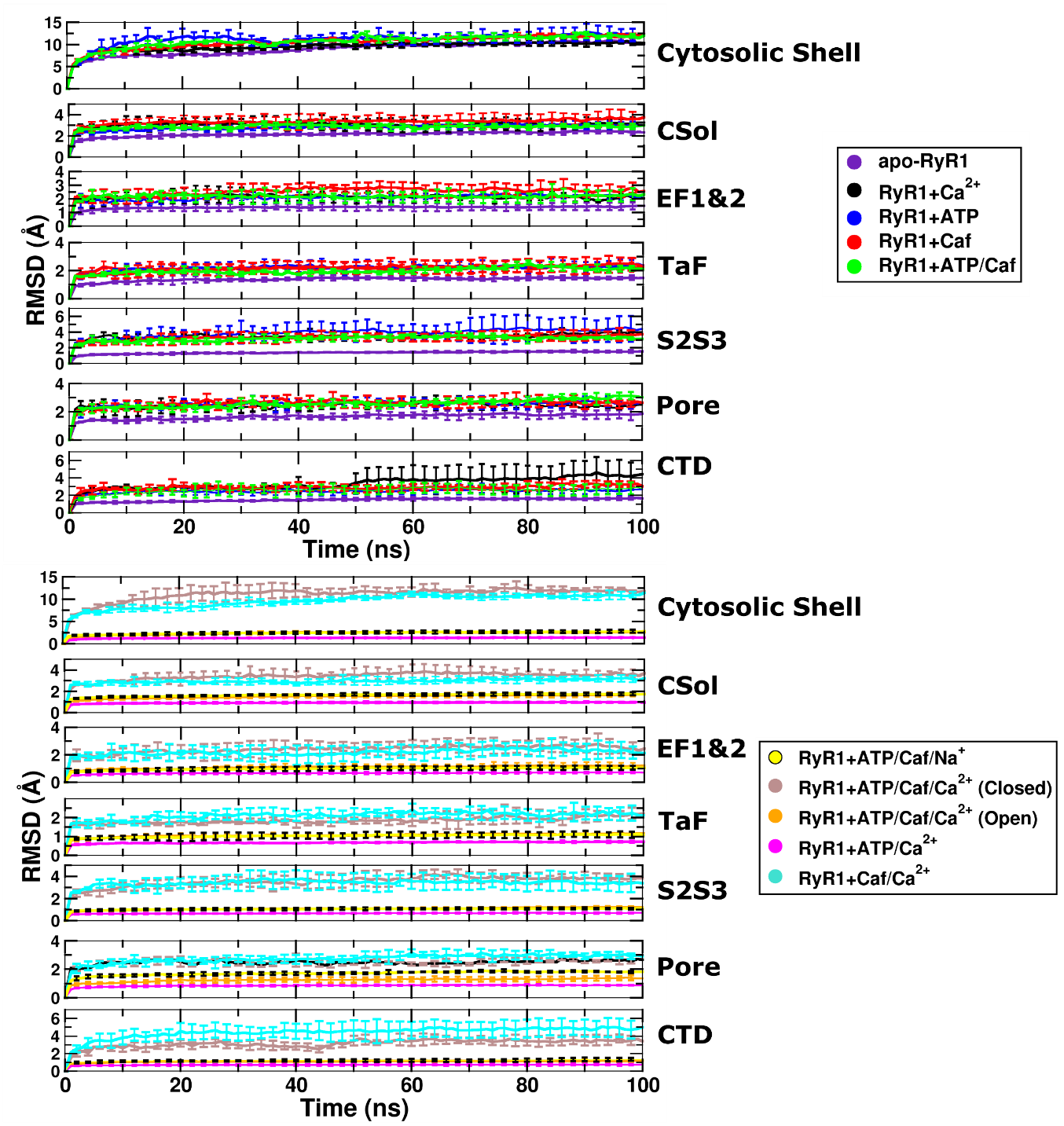


**Fig. S4. Evaluation of domain-wise RMSDs in different RyR1 functional states.** We evaluated and compared RMSDs of cytosolic shell region and individual domains of the activation core among different RyR1 states with respect to simulation time. The data indicated that minor disturbances in activator binding sites, in the absence of specific activators, induce large fluctuations in cytosolic shell region. The absence of either ATP, Caf, or Ca^2+^ in respective binding sites cause large electrostatic perturbations, which instigate structural changes or disturbances in entire protein.


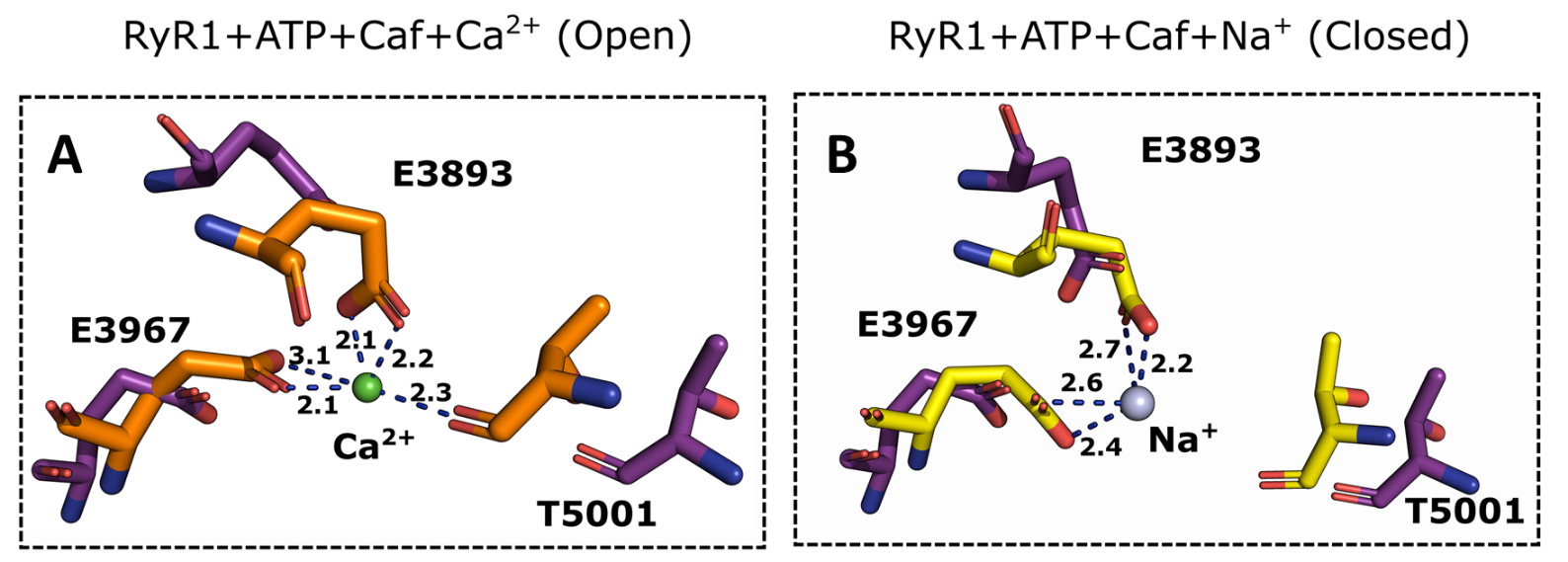


**Fig. S5. Diverse interactions of Na^+^ in Ca^2+^-binding site elucidate its differential pore regulation mechanism.** Structural superposition of Ca^2+^ binding site of apo-RyR1 with (A) Ca^2+^/ATP/Caf occupied open RyR1 and (B) Na^+^/ATP/Caf occupied closed RyR1. Color code: apo-RyR1 in purple, Ca^2+^/ATP/Caf occupied open RyR1 in orange, Na^+^/ATP/Caf occupied closed RyR1 in yellow, Ca^2+^ ion in green, and Na^+^ ion in light blue.


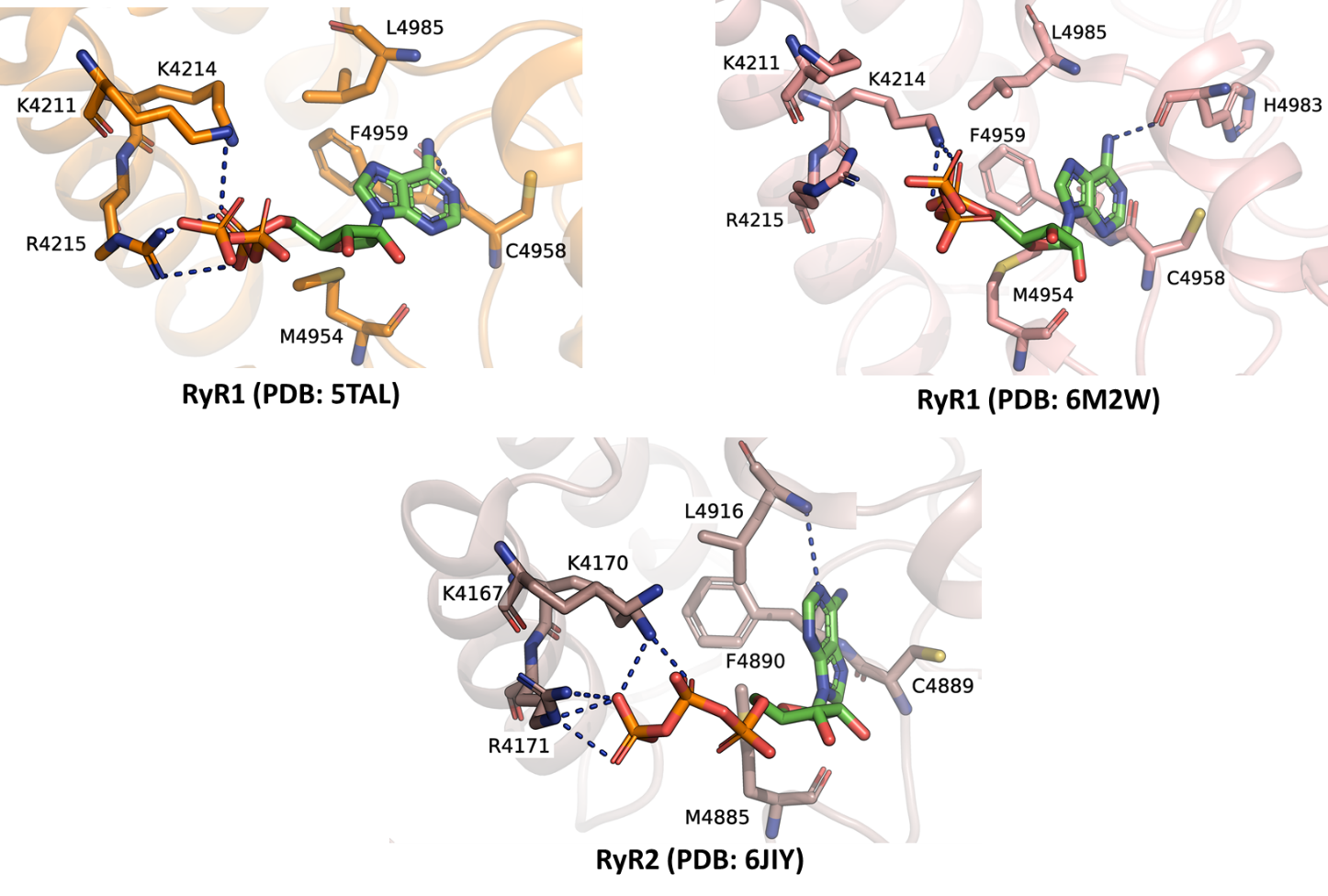


**Fig. S6. Comparison of ATP interactions between RyR1 and RyR2.** We considered two RyR1 conformations (PDB IDs: 5TAL and 6M2W) and one RyR2 conformation (PDB: 6JIY) for the comparative analysis. The comparison of ATP-interactions suggest that ATP binding is identical and interacting residues are well conserved in RyR1 and RyR2. Color code: 5TAL in orange, 6M2W in salmon, 6JIY in brown, and ATP in green. H-bond interactions are shown as blue dotted lines.


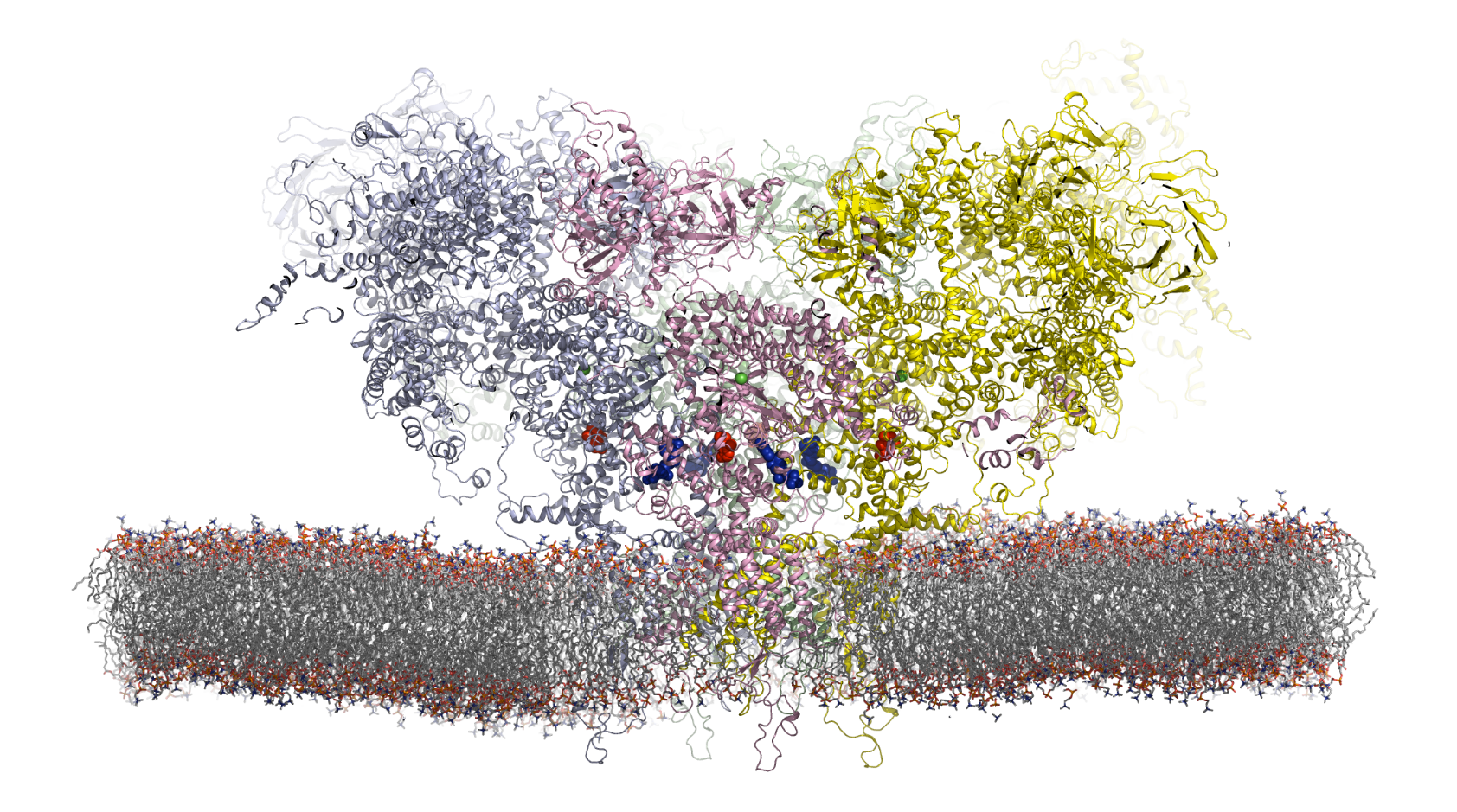


**Fig. S7. Representative snapshot of membrane embedded RyR1 simulation system.** The protein is depicted in cartoon representation with each monomer in different color. Ca^2+^, ATP, and caffeine are shown in van der Waals representation in green, blue, and red respectively. To better visualize the TM part of the protein, we removed lipids masking the TM region. The POPC lipid bilayer is sliced shown in gray stick representation with O atoms in red and N atoms in blue. Water molecules and neutralizing ions were not shown for better clarity.
